# Insight into the Broad Field of Polymer Nanocomposites: From Carbon Nanotubes to Clay Nanoplatelets, via Metal Nanoparticles

**DOI:** 10.3390/ma2042095

**Published:** 2009-11-30

**Authors:** Eduard A. Stefanescu, Codrin Daranga, Cristina Stefanescu

**Affiliations:** 1 Department of Chemical & Life Science Engineering, Virginia Commonwealth University, Richmond, VA 23284, USA; 2 Department of Civil & Environmental Engineering, University of Wisconsin, Madison, WI 53706, USA; E-Mail: daranga@wisc.edu (C.D.); 3 Department of Chemistry, Louisiana State University, Baton Rouge, LA 70803, USA

**Keywords:** carbon nanotube, Laponite^®^, montmorillonite, metal, nanocomposite

## Abstract

Highly ordered polymer nanocomposites are complex materials that display a rich morphological behavior owing to variations in composition, structure, and properties on a nanometer length scale. Metal-polymer nanocomposite materials are becoming more popular for applications requiring low cost, high metal surface areas. Catalytic systems seem to be the most prevalent application for a wide range of metals used in polymer nanocomposites, particularly for metals like Pt, Ni, Co, and Au, with known catalytic activities. On the other hand, among the most frequently utilized techniques to prepare polymer/CNT and/or polymer/clay nanocomposites are approaches like melt mixing, solution casting, electrospinning and solid-state shear pulverization. Additionally, some of the current and potential applications of polymer/CNT and/or polymer/clay nanocomposites include photovoltaic devices, optical switches, electromagnetic interference (EMI) shielding, aerospace and automotive materials, packaging, adhesives and coatings. This extensive review covers a broad range of articles, typically from high impact-factor journals, on most of the polymer-nanocomposites known to date: polymer/carbon nanotubes, polymer/metal nanospheres, and polymer/clay nanoplatelets composites. The various types of nanocomposites are described form the preparation stages to performance and applications. Comparisons of the various types of nanocomposites are conducted and conclusions are formulated.

## 1. Introduction to Polymer Nanocomposites

Highly ordered polymer nanocomposites are complex materials that display a rich morphological behavior because of variations in composition, structure, and properties on a nanometer length scale [1,2,3,4]. Novel physical properties of soft and bulk polymer nanocomposite materials are also dependent on the supramolecular organization of the nanostructures [5]. The presence of the nanoparticle and the interaction of the polymer with the particle, as well as the particle orientation in a dispersed fluid composition may lead to a variety of ordered composite materials in the bulk or film [6,7,8]. Incorporation of metals into polymer matrixes has been shown to produce unique optical, magnetic and dielectric properties at the nano- and macro-scale in nanocomposites, apparently due to the surface and confinement effects of the nanoparticles [9,10,11,12,13,14,15]. The preparation of magnetic nanocomposites that comprise polymer shells and magnetic nanoparticles constitutes a very attractive approach since the modular nature of polymeric materials facilitates the design of a wide range of hybrid nanocomposites of various compositions [10]. Additionally, the inherent dipole moment in ferromagnetic colloids enables the one- and two-dimensional assembly of such materials into novel mesostructures. The development of magnetic assemblies from ferromagnetic and superparamagnetic nanoparticles on supporting surfaces has been demonstrated in the past. Some of the structures that have been shown to occur as a result of magnetic assembly include 1-D chains [16], flux closure rings [17], 2-D superlattices of closed packed nanocrystals [18], and 3-D labyrinth-like suprastructures [19].

During the past decade carbon nanotubes (CNTs) have attracted much attention for their ability to increase electrical conductivity and thermo-mechanical properties of polymers at relatively low content [20,21,22,23,24,25,26,27,28]. For example, much of the literature describing conductive polymer/CNT nanocomposites is mainly focused on decreasing the percolation threshold in order to reduce the cost and improve the physical properties and processability of composites [29,30]. CNTs are characterized by very high aspect ratios, having diameters in the order of a few nanometers and lengths in the range of hundreds nanometers. It is known that, despite their light weight characteristics, CNTs are roughly 10 to 100 times stronger than the strongest steel [21]. One of the main challenges in creating high performance polymer/CNT nanocomposites consists in one’s ability to obtain an optimum dispersion and a proper adhesion of CNTs within the polymer matrix [8,30,31,32,33]. A proper dispersion is often times impossible to achieve with pristine carbon nanotubes because of their small diameter and completely hydrophobic surface [34]. These practical difficulties are most times overcome by functionalizing the surface of CNTs with small polar groups or with pre-polymers that are either identical or structurally similar to the matrix material that needs to be reinforced [35,36]. Various processing methods have been designed to produce polymer/CNT nanocomposites. For example, solution processing can be used if the polymer is soluble in a solvent that is capable of suspending the CNTs [28,35,37,38]. Melt processing is also available for thermoplastic polymers, where the nanotubes are mechanically sheared and compounded in the molten polymer matrix [27,34,39]. Some of the current and potential applications of polymer/CNT nanocomposites include photovoltaic devices, optical switches, electromagnetic interference (EMI) shielding, aerospace and automotive materials, bicycle and tennis racquet frames, packaging, adhesive and coatings [21].

On the other hand, a large number of metals have been utilized in the past to produce polymer composites with distinct characteristics. For example, polyaniline (PANI)/platinum (Pt) nanocomposites are extensively studied for the extraordinary ability of the metal to catalyze redox reactions [40]. Gold (Au) nanoparticles have attracted considerable attention for their catalytic properties towards carbon monoxide and methanol oxidation [41]. Palladium (Pd) and its polymer-based composites represent an important catalytic class for the formation of C-C bonds, while exhibiting tolerance for several functional groups such as carbonyl and hydroxyl [42]. Cobalt (Co) is a versatile ferromagnetic metal used in the fabrication of magnetic, wear-resistant alloys and composites [10,12,43,44]. Furthermore, nickel (Ni) is a magneto-selective material characterized by negative magneto-striction [45,46]. Nanocrystalline Ni/polyvinylidene fluoride (PVDF) composites exhibit a high dielectric constant at very small metal concentrations [14]. In addition, silver (Ag) particles characterized by spherical shapes exhibit a very strong absorption band in the visible region due to their surface plasmon resonance [47]. Owing to their extraordinary characteristics, polymer/metal nanocomposites can be utilized for the design of several advanced devices that include optical limiters and polarizers, optical sensors, magneto-optical modulators, electro-optical filters, superparamagnetic materials, electro-catalytic electrodes, chemical absorbers, and so on [20].

In addition to metal- and CNT-composites, in recent years a broad literature has emerged that examines the fundamental relationships between network structure, chain dynamics, ionic conductivity and dimensional stability, in cross-linked nanosized polymer-clay networks [5,48,49,50,51,52,53,54]. Perhaps the two most discussed clays in literature are Laponite^®^ and montmorillonite. Laponite^®^ clay is an inexpensive and environmentally benign disc-shaped silicate with a plate diameter of 25–30 nm and a thickness of approximately 1nm [54,55]. In aqueous solutions and gels hydrophilic polymers strongly adsorb to the charged Laponite^®^ nanoparticles leading to the formation of transparent systems. Smectites such as montmorillonite are 2:1 charged phyllosilicates that contain exchangeable interlayer cations and show the ability to intercalate various polymers [6]. Montmorillonite clay produces an opaque suspension of predominantly exfoliated platelets that range on average in size from ca. 70 to 100 nm across and are approximately 1 nm thick [5]. When dispersed alone in water such clays exhibit a Newtonian behavior, but in the presence of polymers the interaction between the polymer chains and the particles causes a major change in the rheological behavior of dispersions [56]. Flow birefringence studies demonstrated that upon shear the clay platelets orient along the flow direction [3,4]. In solution these clay particles can only adsorb a maximum amount of polymer until all the clay surfaces are covered [1]. The polymer and clay build a network-like structure which is interpenetrated by a sub-network of interconnecting pores containing excess polymer and water. During the layer by layer film preparation method the exfoliated solution-structure collapses, reorders and re-intercalates into blob-like chains and layers [5].

The aim of the present paper is to provide an extended review of the recent scientific methodologies utilized to prepare and characterize polymer nanocomposites containing metals, carbon nanotubes and clay nanoplatelets as reinforcing agents. Keeping in mind the importance of nanotechnology, our efforts were concentrated on gathering information from some of the best journals in the area of polymer composites, with the purpose of helping the reader stay up-to-date with the current cutting-edge developments in the field. The reader is cautioned, however, that although the review contains a great deal of information, it is far from being an exhaustive source of information in this matter.

## 2. Polymer/Carbon Nanotubes Nanocomposites

Following the recent developments in the fields of nanotechnology, carbon nanotube (CNT) reinforced polymer composites received tremendous attention in the past decades, and currently occupies an elite place among the most studied polymer composites. When properly dispersed within polymer matrixes, CNTs have the ability to improve the properties of the resultant materials several orders of magnitude relative to the unfilled polymers. The enhanced properties may include tensile behavior, strength, toughness, stiffness, electrical and thermal conductivity and crystallization kinetics. The focus of the present section is to bring together, in a coherent way, various aspects of the recently published research in the field of polymer/CNT nanocomposites. The polymer matrixes that are covered in this section are among the most researched and utilized in academia and industry, and include polystyrene, poly(ethylene oxide), poly(ε-caprolactone), polypropylene, various nylons and poly(ethylene terephthalate).

### 2.1. Polystyrene/Carbon Nanotubes Composites

Xu *et al.* used an electrostatic assembly to synthesize polystyrene (PS)-carbon nanofibers (CNF) via a bottom-up method [57]. The authors mixed a home-made PS latex with an aqueous suspension of oxidized CNF, and obtained the nanocomposites through the electrostatic interaction of the cationic PS with the anionic CNF. They claim that the molding temperature has an important effect on the morphology and electrical conductivity of PS-CNF composites, where the optimal temperature and pressure of 185 °C and 25 MPA, respectively, result in a percolation threshold below 2 wt%. Thermal analysis measurements showed that the presence of the CNF increases the onset and the thermo-oxidative temperature by 60 °C. However, the CNF do not alter the glass-transition temperature (*T*_g_) of the polymer. Similar results were obtained by Wang *et al*., who prepared a composite of syndiotactic PS (sPS) filled with carbon nanocapsules (CNC) using a solution-blending method coupled with ultrasonication [58]. Just like Xu *et al.*, they observed that the filler addition had no impact on the Tg of the polymer, but that it led to an increase in the melt-crystallization temperature of the β–form sPS. In addition, the presence of the filler triggered a fast crystallization of the sPS, induced either by melt-quenching or by slow cooling of the molten state, which was further attributed to the existence of a profound primary nucleation density. The authors concluded that CNCs are excellent nucleating agents that improve the crystallinity and thermal stability of sPS. Likewise, Shen *et al.* used CNFs as nucleating agents to produce PS microcellular nanocomposite foams with uniform size distributions [59]. They found that small amounts of CNFs could drastically reduce the composite’s cell dimensions and increase the cellular density. The favorable surface and geometrical characteristics of CNFs were found to be the main reasons for the excellent nucleation efficiency of the filler.

On the other hand, combining polystyrene with single-wall carbon nanotubes (SWCNT) Mu *et al.* produced electrically conductive composites with good rheological and mechanical properties [60]. In short, they created a contiguous SWCNT cellular-structure by coating PS pellets with SWCNTs and pressing them afterwards at elevated temperatures. When compared to nanocomposites with well-dispersed SWCNTs, the novel SWCNT cellular-structures proved to be better electric conductors, showing a lower electrical percolation threshold. Such PS composites having the SWCNTs well dispersed in the polymer matrix are described by Chang *et al.* [61]. The authors varied the concentration of SWCNTs and analyzed the morphologies, as well as the electrical and mechanical properties, of the resultant nanocomposites. They observed that composites that were subjected to an annealing treatment exhibited higher electrical conductivities than the raw composites. However, even after annealing, the SWCNT/PS composites described by Chang *et al.* displayed lower electrical conductivities than the PS/SWCNT cellular-structures described by Mu *et al.* It has been suggested that, in general, it is difficult to disperse SWCNTs in polymers since the energy of mixing is endothermic up to nanotube diameters of 2.2 nm [62]. Furthermore, using a “grafting to” technique Xie *et al.* prepared composites of SWCNTs with styrene copolymers [63]. In their method the reaction occurred at relatively mild conditions between the alkyne groups present on the SWCNTs and the benzyl chloride groups from the styrene copolymers. This coupling reaction significantly improved the bonding density at the polymer/SWCNT interface, with an overall efficiency of 81 wt% as revealed by thermogravimetric analysis (TGA). The authors claimed that the steric hindrance created by the polymer, which was physically adsorbed at the surface of SWCNTs, limited the number of cross-links between individual SWCNTs. At last, Putz *et al.* studied the effect of interfacial interactions in functionalized SWCNT/PS nanocomposites and found that for concentrations less than 0.1% of SWCNTs the elasticity of the composites remained comparable to the one of pure PS [64]. As the concentration of SWCNTs increased, however, a more pronounced elastic behavior of nanocomposites was observed. The authors correlated their findings with theoretical calculations and found significant inconsistencies in the resultant values.

Often times highly conductive polymer/CNT composites lose their conductivity after melt processing. Using multi-wall carbon nanotubes (MWCNT) and PS, Cipriano *et al.* designed an efficient route to obtain highly conductive extruded plastics, of which conductivity could be recovered after processing through annealing at temperatures higher than Tg of the polymer [65]. The authors suggested that the conductivity recovery involved a transition of the unconnected particles, which were aligned in the flow direction during extrusion-processing, to an interconnected network after annealing. In line with this idea, they performed a series of electrical measurements on commercial polydisperse polystyrene PS/MWCNT composites and observed very high conductivities (ca. 1 S/m) at a filler loading of 8 vol% [66]. The same group studied the impact of the aspect ratio of MWCNTs on the rheology and flammability of extruded PS/MWCNT nanocomposites and found that MWCNTs with larger aspect-ratios triggered higher storage moduli and complex viscosities in nanocomposites [67]. Additionally, the larger aspect-ratio nanocomposites were more efficient at reducing flammability, since they resulted in a reduced mass-loss of the composites subjected to combustion.

**Figure 1:**
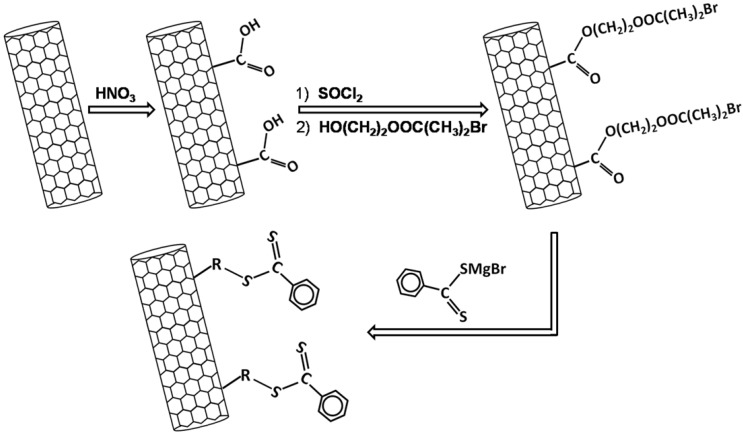
Schematic showing the steps used by Cui *et al*. to synthesize nanotube-attached RAFT agents.

Cui *et al.* used reversible addition fragmentation chain-transfer (RAFT) to synthesize polymer-connected-MWCNT nanocomposites by grafting a RAFT agent, and subsequently PS chains, on the surface of MWCNTs [68]. Figure 1 describes the steps used by the authors to prepare the nanotube-attached RAFT agents. After being synthesized, the nanotube-attached RAFT agents were further reacted with the styrene monomers in the presence of AIBN and THF to obtain the final polymer composites. The grafting of the PS chains to the surface of the MWCNTs led to the formation of core-shell structures. The authors claim that their method can be used to synthesize other polymer/CNT systems, including composites based on copolymers of acrylate and styrene-type monomers. In fact two years after their initial report the same group synthesized a composite of MWCNT and poly(methylmethacrylate)-block-polystyrene (PMMA-b-PS) following a similar route [69].

### 2.2. Poly(Ethylene Oxide)/Carbon Nanotubes Composites

Chatterjee *et al.* have prepared and characterized poly(ethylene oxide) (PEO) nanocomposites containing SWCNTs [33]. The authors used a lithium based surfactant to improve the dispersion of SWCNTs in the polymeric matrix. Their composites exhibited an electrical percolation of 0.03 wt% and a geometrical percolation of 0.09 wt%. Inside the polymer matrix the SWCNTs were found to be either under tension or strongly interacting with the PEO-surfactant complex. At very small loadings (ca. 0.2 wt%) the filler was capable of significantly lowering the PEO crystallinity and crystallization temperature. Furthermore, the same group studied later, via rheological measurements, the response of fractal networks in SWCNT/PEO nanocomposites [32]. They found that the composites exhibited a strong shear-thinning behavior. Additionally, for systems corresponding to the semidilute regime the authors observed that the constant rate shear triggered a stress response that went through a maxima and decayed to steady values at long times. They attributed the stress maxima to the SWCNT network superstructure, since the molecular weight (Mw) of the PEO used was too low to trigger a chain entanglement that would result in a stress overshoot. Single-wall carbon nanotubes have been also used as fillers in amphiphilic cross-linked PEO-fluoropolymer networks [70]. Prior to the dispersion of filler in the polymer matrix the surface of SWCNTs was functionalized with diamine-terminated polyethylene glycol (PEG) chains. As anticipated, the surface functionalization led to an improved dispersion of the treated nanotubes in the polymer matrix, compared to the non-treated ones, which was attributed to an enhancement of the non-covalent interactions between the filler and the polymer. In turn, the improved dispersion resulted in a greater reinforcing effect, where the tensile modulus (E) of the composite was increased by 430% relative to the unfilled material.

Similar to the case of SWCNTs, various surface treatments have been also applied to MWCNTs to improve their reinforcing effects in the PEO matrix [26,71,72]. Song observed that acid-treated MWCNTs had a significantly higher effective volume fraction than the real volume of the untreated MWCNTs [26]. This effect triggered an improvement in the rheological response of the composites filled with acid-treated MWCNTs. In contrast, the nanotubes treated with long, amine-terminated, alkyl chains (e.g. octadecylamine) did not make a significant impact on the viscosity and modulus of resultant composites, as observed through comparison with unfilled PEO [26]. Jin *et al.* studied the crystallization behavior of PEO nanocomposites, containing modified and unmodified MWCNTs, by means of DSC measurements [72]. The chemically modified MWCNTs contained either carboxylic or hydroxyl functional groups. The results suggested that incorporating either the treated or the untreated MWCNTs generated a decrease in the polymer crystallinity, similar to what has been observed by Chatterjee *et al.* for SWCNTs [33]. In short, the filler decreased the nucleation sites in PEO nanocomposites, but led to an increase in the spherical crystal size compared to neat PEO [26]. Moreover, to improve the properties of PEO/MWCNT nanocomposites, Yang *et al.* have functionalized the nanotubes by reacting the carboxylic acid groups from the filler’s surface with the glycidyl terminal groups of poly (bisphenol-A-co-epichlorohydrin) [71]. The grafting of phenoxy chains onto MWCNTs improved the dispersion of the filler in the polymer matrix owing to the high affinity of PEO and phenoxy chains for each other. In turn, the improved dispersion caused the enhancement of several properties of the composites, such as stiffness, strength, ductility, and toughness. As expected, an increase in the amount of MWCNTs in the composites resulted in a decrease in the size of the PEO spherulites [71].

At this point it should be noted that the technique known as *“solution-sonication”* represents a very common way to prepare PEO/MWCNT nanocomposites, and that the method is often used by various scientific research-groups [24,25,26,72,73,74]. Using this technique, Narh *et al.* studied the impact of agglomerated versus non-agglomerated MWCNTs on the thermo-mechanical properties of PEO/MWCNTs [25]. The authors found that the breaking-strain values, during mechanical measurements, were much lower for the nanocomposites than for the unfilled polymer. The resultant breaking-strain values were slightly higher for the composites containing non-agglomerated MWCNTs than for the agglomerated counterparts. The tensile modulus of nanocomposites, however, was observed to be 110 times higher than the one of the unfilled PEO. Furthermore, Song studied the rheological properties of PEO/MWCNT nanocomposites as a function of filler loading, and found that the shear viscosity of composites increased when the filler content was increased [74]. Complimentary, oscillatory shear experiments revealed that an increase in the filler loading generated a more pronounced solid-like behavior of the composite dispersion, owing to stronger polymer-particle and particle-particle interactions. Similarly, Abraham *et al.* observed an increase in the rheological properties with increasing the amount of MWCNTs [24]. The electric conductivity was also observed to improve with the filler addition. However, the increase in MWCNT loading induced a prominent decrease in the crystallization temperature of the PEO in the solid composites.

**Figure 2:**
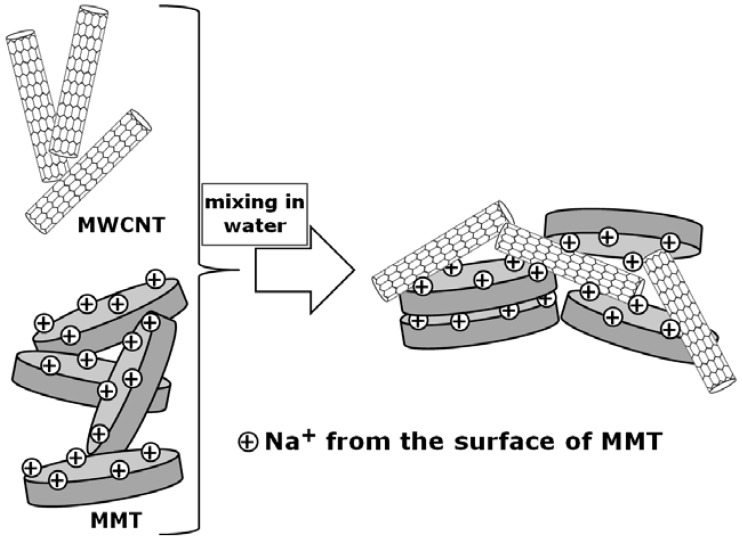
Schematic showing the interaction between MWCNTS and MMT nanoplatelets in water.

Finally, Wang *et al.* proposed a method for the preparation of PEO nanocomposites containing multiwall carbon nanotubes/montmorillonite (MWCNT/MMT) hybrids [73]. The authors developed their technique relying on the formation of stable MWCNT/MMT aqueous dispersions, which are typically caused by the strong interactions between MWCNTs and MMT platelets. As indicated in Figure 2, in water the negatively charged surface of MWCNTs can interact with the Na cations from the surface of MMT platelets. This interaction was the key for the formation of stable MWCNT/MMT aqueous dispersions. The PEO composites containing the MWCNT/MMT hybrids exhibited high mechanical properties, owing to the formation of an extended network structure in the ternary system [73].

### 2.3. Poly(ε-Caprolactone)/Carbon Nanotubes Composites

Poly(ε-caprolactone) (PCL) is a biodegradable polymer that has been intensively studied over the past decades for its potential applications as implantable biomaterial. There are several reports in literature describing addition of either SWCNTs [8,31,75] or MWCNTs [22,23,76,77,78,79,80] to PCL to improve its physical properties. PCL/SWCNT composites have the unique advantage of combining the lightweight of these materials with their enhanced mechanical, thermal and electrical properties. Mitchell *et al.* prepared PCL/SWCNT nanocomposites with the aid of a zwitterionic surfactant (12-aminododecanoic acid) (ADA) [31]. Owing to the ability of surfactant to interact with both SWCNTs and PCL, an excellent dispersion of the filler within the polymer matrix was achieved. As a result, the nanocomposites exhibited geometrical and electrical percolations at SWCNT loadings of only 0.08 wt%. Furthermore, the same research group also reported on the ability to control the nano- and meso-scale arrangement of polymer chains in PCL/SWCNT composites containing the same zwitterionic surfactant (ADA) [8]. It was found that the SWCNTs, which exhibited an effective aspect ratio of *ca*. 750, were able to act as nucleators for the crystallization of PCL. In consequence, the polymer’s rate of crystallization increased a hundred fold in the presence of filler. Additionally, the authors subjected the nanocomposites to uniaxial extension and observed, using Raman spectroscopy, a strong alignment of SWCNTs within the polymer matrix. At last, Priftis *et al.* reported a novel approach in which they functionalized SWCNTs (along with MWCNTs) with a titanium alkoxide catalyst and used the modified filler to surface-initiate the coordination polymerization of ε-caprolactone monomer [75]. Their results proved that the surface grafted catalyst was able to effectively promote the polymerization of monomer from SWCNTs, leading to the formation of PCL/SWCNT nanocomposites.

Ring opening polymerization (ROP) of ε-caprolactone monomer represents a very utilized method to fabricate PCL/MWCNT nanocomposites [22,36,81]. Chrissafis *et al.* used *in situ* ROP to fabricate PCL composites containing MWCNTs, along with other fillers such as fumed silica and montmorillonite [22]. The tensile strength of nanocomposites was observed in all cases to be higher than the one of neat PCL. Thermal analysis revealed that MWCNTs could decelerate the thermal degradation of PCL owing to a shielding effect introduced by the filler. In contrast, fumed silica and modified montmorillonite were found to accelerate the polymer’s degradation due to chemical reactions that these fillers could induce in the PCL matrix. All fillers were observed to affect the activation energy of decomposition, but none of them could alter the decomposition mechanism of PCL [22]. Furthermore, Castro *et al.* grafted PCL chains on the surface of carbon nanotubes (CNT), via a ROP approach, and tested the vapor sensing properties of the resultant nanocomposites [81]. For that, the authors prepared conductive polymer composite transducers utilizing a layer-by-layer approach. The resultant PCL/CNT transducers were able to provide quantitative and discriminating responses after exposure to different polar and non-polar chemical vapors, such as water, methanol, toluene, tetrahydrofuran and chloroform. Finally, Wang *et al.* functionalized carbon nanofibers (CNF) with PCL chains by *in situ* ROP in the presence of stannous octoate [36]. The authors claim that the content of polymer grafted to the filer’s surface could be controlled by adjusting the feed ratio of monomer to hydroxyl-functionalized CNFs. The highest polymer amount that they were able to graft was around 64 wt%.

Another effective way to produce PCL/MWCNT nanocomposites is the melt mixing of constituents [23,78,80]. Using such a method, Thomassin *et al.* fabricated PCL composites containing MWCNTs of two different diameters, aiming to improve the electromagnetic interference (EMI) shielding characteristics of the polyester [23]. Although the thin MWCNTs were found less prone to break down during melt mixing than thick MWCNTs, the use of thick MWCNTs triggered a more significant improvement of mechanical and electrical properties in the resultant composites. As a result of breaking-down, thick MWCNTs generated electrical conductivities higher than 1 S/m at fairly low loadings (0.7 wt%), inducing very good EMI shielding properties in the material [23]. Also using a melt compounding method Xu *et al.* prepared a series of PCL/MWCNT nanocomposites, and studied their crystallization kinetics and mechanical behavior [80]. The authors observed that the presence of MWCNTs, even in very small amounts, triggers a significant increase of the shear moduli of the composite compared to neat PCL. Since the incorporation of MWCNTs accelerated the nucleation and crystal growth of PCL, the filler loading was found to critically affect the degree of crystallinity and the time of crystallization in composites. The authors claim that the activation energy of crystallization in composites was higher for small MWCNT loadings and it decreased gradually as the filler loading was increased [80]. Moreover, Wu *et al.* blended PCL with polylactide (PLA) and coupled the resultant blend with functionalized MWCNTs via melt mixing [78]. The authors observed that the carboxylic MWCNTs were predominantly dispersed either in the PCL phase or at the interface between the two polymeric phases, but not too much in the PLA phase. The positioning of the filler at the interface resulted in an enhancement of the phase morphology of the ternary system, owing to a superior MWCNT-mediated interfacial adhesion. In consequence, rheological and electrical percolation threshold values of the ternary composites resulted lower than the ones corresponding to the PCL/PLA systems.

**Figure 3:**
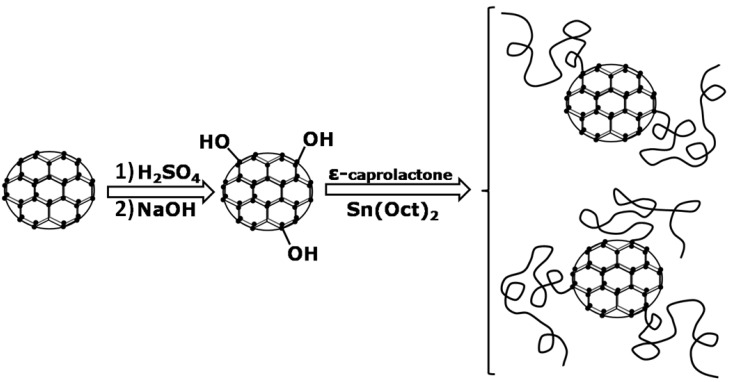
Schematic displaying the steps needed to obtain 2 and 3 arm PCL-grafted-fullerene.

In addition to incorporating carbon nanotubes in polymers, some effort has been recently devoted to the addition of carbon nanospheres, also known as fullerenes or buckyballs, to polymers [82,83,84]. Kai *et al.* synthesized, via ring opening polymerization of ε-caprolactone, star shaped PCL composites containing a fullerene core [84]. As presented in Figure 3, the fullerenes’ surface was functionalized with hydroxyl groups prior to the polymerization of ε-caprolactone. Conducting bulk polymerizations the authors obtained fullerene cores having 2 and 3 arms, and they estimated the ratio of the two structures to be 1:1.

As opposed to this case, polymerization of ε-caprolactone in toluene resulted in fullerene cores having predominantly three arms attached. The same group later developed a method to produce PCL/fullerene nanocomposite films [82]. Within such a composite film the fullerene domains were found to aggregate and form microscopic fullerene particles with an average diameter of ca. 2 µm. Furthermore, the authors argued that the microscopic aggregates impacted the polymer crystallinity via two major effects: a confinement effect and a nucleating effect. Owing to these two effects the crystallization temperature of the composites was found to be higher than that of the neat polymer. Additionally, the final crystallinity of the PCL in the composite was lower than the one of the unfilled polymer. Finally, the authors found that in fullerene-capped PCL composites the mechanical properties were strongly dependent on the fullerene loading and on the end-capping type (*i.e.,* single *vs*. double) [83].

### 2.4. Polypropylene/Carbon Nanotubes Composites

Owing to its low price and excellent physical properties, polypropylene (PP), and more specifically isotactic polypropylene (iPP), is one of the most extensively studied thermoplastics, with widespread applications such as home appliances and automotive construction [85,86]. Probably the most convenient and efficient way to produce PP/CNT composites is represented by melt compounding of components [87,88,89]. Using such a method, Pujari *et al.* prepared and characterized a set of MWCNT dispersions in PP [88]. Additionally they prepared a second set of dispersions employing a solid-state shear pulverization method, and compared the composites with the ones obtained through melt mixing. The authors observed that the nanocomposites prepared via melt compounding exhibited slower crystallization kinetics and lower mechanical stiffness than the shear pulverized samples. However, the melt mixed samples showed higher electrical conductivity. In addition, as opposed to the melt mixed samples, the pulverized composites showed no measurable low elastic plateau in melt rheology [88]. Furthermore, Hou *et al.* investigated the structural orientation and tensile properties of extrusion elongated PP/MWCNT melts [87]. The authors adjusted the orientation level in the resultant composite sheets by altering the draw-speeds after extrusion. By utilizing fast draw-speeds on nanocomposites with high MWCNT loadings, they were able to optimize simultaneously the strengthening and the toughening effects in the samples. The authors also noticed that although the MWCNTs acted as nucleators for the polymeric crystalline superstructure, the filler did not modify the orientation degree of the crystalline lamellae.

Wu *et al.* employed a maleic anhydride (MA) grafted polypropylene to prepare PP/MWCNT nanocomposites via melt mixing [89]. The MA grafts played the role of compatibilizers. The authors showed that while in the absence of MA the filler was only dispersed at the microscale within the PP matrix, addition of MA triggered the nanoscale dispersion of MWCNTs. It was further observed that the chain motion of the bulk polymer was not strongly affected by the presence of MWCNTs, and that the effective diffusivity of the composites was very similar to the one of the neat PP. Moreover, Xu and Wang investigated the impact of MWCNT aspect-ratio on the normal stress differences in iPP/MWCNT melts [90]. The aspect ratio of the nanotubes utilized was in the range of 22 to 45. At high MWCNT loadings the authors observed the occurrence of a non-equilibrium MWCNT network structure. A strong impact of the filler aspect ratio on the composites rheology was detected by means of die-swell measurements. While the composites containing large aspect-ratio MWCNTs exhibited die-shrinkage, the composites containing short MWCNTs showed a rather strong die-swelling effect [90].

Besides studying the rheological properties of composites, researchers often investigate the ability of fillers to act as nucleating agents for the polymer crystallization [85,86,87]. Liu *et al.* studied the nucleating ability of halloysite nanotubes (HNTs) towards crystallization kinetics of iPP [85]. Apparently, HNTs trigger predominantly the formation of the trigonal β-crystal iPP in the detriment of the monoclinic (α) and orthorhombic (γ) crystal geometries. The authors stated that the occurrence of the β-crystal iPP was strongly influenced by the HNT loading, and showed that composites with 20 phr (parts per hundred rubber) HNT had the highest β-crystal content. Additionally, the authors claimed that a decrease in the cooling rate during non-isothermal crystallization could lead to an increase of the β-crystal iPP content, with a maximum obtained at a cooling rate of 2.5 °C/min. When isothermal crystallization was employed, the occurrence of β-crystal iPP was detected between 115 – 140 °C. The authors attributed the formation of the β-crystal iPP to the unique surface characteristics of HNTs [85]. Finally, Lu *et al.* investigated the influence of SWCNTs and MWCNTs on the crystallization behavior of iPP [86]. The authors observed that when iPP was crystallized from quiescent melt both SWCNTs and MWCNTs exhibited a strong nucleating effect that led to the formation of a transcrystalline layer of ordered iPP lamellar crystals in the vicinity of the fillers. Contrary to what was detected for the halloysite nanotubes [85], CNTs were observed to induce the formation of monoclinic α-crystal iPP exclusively [86]. The authors attributed the strong alignment of the transcrystalline layer around the nanotubes surface to the fact that iPP macromolecules started wrapping around the CNTs in the melt, which triggered a melt confinement that was maintained during the nucleation process.

### 2.5. Nylon/Carbon Nanotubes Composites

Nylon 6, or polyamide 6, is typically obtained through the ring opening polymerization of the cyclic caprolactam monomer, but it can also be synthesized through the step polymerization of aminocapronic acid. Several reports in literature talk about the incorporation of carbon nanotubes in nylon 6 to improve its physical properties [21,29,30]. Jose *et al.* synthesized through an electrospinning method nanofibrous composites of nylon 6 and MWCNTs [21]. Carboxylic acid functional groups were attached to the surface of MWCNTs prior to nanocomposite preparation. The diameter of the electrospun nanocomposite fibers was observed to decrease at higher MWCNT loadings. The authors also noticed a transformation of the γ-phase crystals of nylon 6 into a mixture of α- and γ-phase crystals as the collector speed increased. Additionally, a significant alignment of the MWCNTs parallel to the main axis of fibers was detected [21]. Furthermore, Brosse *et al.* investigated the effect of MWCNTs on the lamellae morphology of nylon 6 [29]. The authors observed that when MWCNTs were well dispersed in the polymer matrix, through melt compounding, the crystalline lamellae of nylon 6 grew with a perpendicular alignment to the surface of nanotubes. Although the authors claim that the MWCNT-induced epitaxial growth is particular to polyamide 6, this feature was also noticed by Lu *et al.* for PP/MWCNT composites [86]. As the dispersion of the filler within the nylon 6 matrix was further improved, an increase in the amount of polymeric transcrystalline epitaxial crystallites could be detected. Li and Shimizu prepared hierarchically structured nanocomposites by dispersing MWCNTs in a poly(vinylidene fluoride) (PVDF)/nylon 6 matrix via high shear processing [30]. The authors observed an unexpected positioning of the filler exclusively in the nylon 6 phase, forming domains with sizes of 10 to 150 nm that were dispersed within the PVDF phase. They further attributed the high electrical conductivity and ductility characteristics of composites to the existence of nylon 6 nanodomains uniformly distributed in the PVDF matrix.

Besides nylon 6, another highly valued polyamide is nylon 6-6, which is typically obtained through the condensation polymerization of hexamethylene diamine with adipic acid. Incorporation of CNTs in nylon 6-6 has also been reported in recent years [91,92]. Using SWCNTs, Haggenmuller *et al.* prepared nylon 6-6 composites via an *in situ* interfacial polymerization [91]. Prior to nanocomposite preparation, SWCNTs were subjected to either purification or to alkyl-chain functionalization. In addition to preparing the two distinct sets of composites containing either the purified or the functionalized nanotubes, the authors fabricated a third type in which a surfactant (sodium salt of dodecylbenzenesulfonic acid) was added along with the SWCNTs. Although both the functionalization and the surfactant stabilization enhanced the filler dispersion in the solvent, only the functionalized nanotubes achieved a uniform dispersion within the polymer matrix. The other two types of composites, containing purified and surfactant-stabilized fillers, exhibited nanotubes agglomeration, and in consequence, an unsatisfactory dispersion degree. When nanocomposites were subjected to low shear forces nanotubes agglomeration was detected in all cases [91]. Furthermore, Li *et al.* studied the morphology and crystallization behavior of nylon 6-6/MWCNT nanocomposites using nanotubes that were functionalized with poly(hexamethylene adipamide) prior to composite fabrication [92]. The authors detected a very good dispersion of the filler within the polymer matrix, through optical and electron microscopy measurements. Additionally, several melting peaks were observed in DSC measurements, which were attributed to changes in the lamellar thickness of crystallites upon heating. The authors concluded that, on one hand the MWCNTs provided heterogeneous nucleation sites for the polyamide crystallization, but on the other hand the filler network hindered the growth of large crystals.

**Figure 4:**
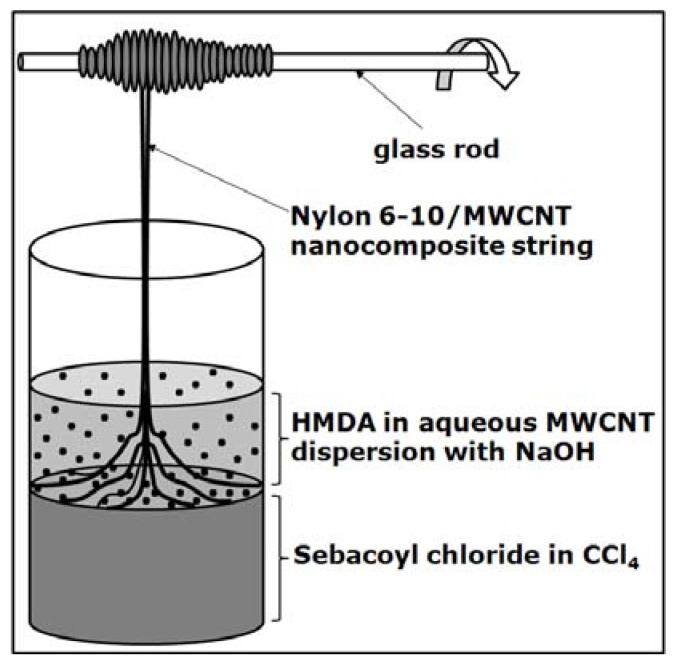
Schematic showing the preparation of nylon 6–10/MWCNT composites via *in situ* interfacial polycondensation of co-monomers.

Other polyamide types were also used to fabricate CNT-based composites, such as Nylon 6-10 [93], nylon 10-10 [94], or nylon 12 [95] following various approaches. One very simple method to prepare nylon/CNT composites is exemplified by Kang *et al.* for polyamide 6-10 (see Figure 4) [93].

The method consisted in the *in situ* interfacial polycondensation of co-monomers (hexamethylene diamine, HMDA, and sebacoyl chloride) dissolved in two immiscible liquid phases (one of which contained also the MWCNTs), followed by the separation of the polymeric string with the help of a glass rod. Theoretically, the method can be applied for the synthesis any other nylon/CNT composites in which the starting co-monomers dissolve in immiscible liquids. Regardless of the type of nylon used by various researchers, all nanocomposites exhibit physical properties superior to the ones of the neat polymers. Generally, an excellent dispersion of the filler leads to good mechanical properties of composites, fact that is attributed in part to the ability of carbon nanotubes to act as nucleation sites in the polymer crystallization process [93,94,95].

### 2.6. Poly(Ethylene Terephthalate)/Carbon Nanotubes Composites

Poly(ethylene terephthalate) (PET) is a widely known semicrystalline thermoplastic polymer that is characterized by excellent physico-mechanical properties, such as high stiffness and strength, and by very good chemical and heat resistance [27,35,39,96,97]. One effective way to prepare PET/CNT composites is represented by *in situ* polymerization of co-monomers in the presence of MWCNTs [96,97]. Using such an approach Antoniadis *et al.* prepared a series of PET/MWCNT composites through a two stage melt-polycondensation [96]. The authors observed that an increase in the filler content triggered a decrease of the intrinsic viscosity of composites. They attributed this behavior to branching and crosslinking of macromolecules owing to specific chemical reactions of nanotubes with co-monomers and/or polymer chains. In addition, the polymer crystallinity in the nanocomposites was observed to decrease with increasing the filler loading. While concentrations up to 1 wt% MWCNTs were found to improve the polymer crystallization rate, loadings of 2 wt% decreased the rate on the basis of chain crosslinking. The authors also found that the MWCNTs acted as nucleating agents for PET crystallization, in agreement with what was observed by other researchers for other polymer/CNT systems [96]. Furthermore, Mun *et al.* used MWCNTs functionalized with (2-hydroxyethyl) triphenyl phosphonium bromide to prepare PET nanocomposites through an *in situ* polymerization approach [97]. After polymerization, nanocomposite fibers were fabricated via a melt spinning approach. A significant improvement in the tensile strength of the polymer was detected after addition of only a small portion of functionalized MWCNTs (0.5 wt%). For MWCNT concentrations between 0 and 1.5 wt%, a direct proportionality was proved to exist between the composites modulus and the filler loading. In addition to the improvement in mechanical characteristics, the thermal properties of nanocomposites were superior to the ones of neat PET [97].

Another valuable method to produce PET/CNT composites is the melt compounding of components, approach that has been also used extensively to prepare other types of polymer composites too [27,34,39]. Yoo *et al.* utilized the melt extrusion method to fabricate PET composites containing MWCNTs functionalized with either benzyl isocyanate or phenyl isocyanate [34]. The mechanical properties (tensile strength and modulus) of composites containing MWCNTs functionalized with isocyanate groups were found superior than in composites containing pristine or acid treated fillers. The authors attributed this behavior to the π – π interactions between the isocyanate groups on the surface of nanotubes and the six member aromatic rings from the PET structure. Additionally, the π–π interactions triggered an improved dispersion of the functionalized MWCNTs in the polymer matrix relative to the unmodified fillers. Interestingly, higher crystallinities were detected for the composites containing the well dispersed functionalized MWCNTs than for the counterparts containing the pristine fillers [34]. Moreover, Averett *et al.* investigated the effects of fatigue and residual strain on the mechanical behavior of PET microfibers reinforced with vapor grown carbon nanofibers (VGCNF) [27]. The authors prepared the composite fibers, with diameters of ca. 25 ± 2 μm through a melt mixing procedure, and compared their resultant properties with the ones of non-reinforced PET fibers (diameters 24 ± 3 μm). The PET composite fibers contained roughly 5 wt% VGCNFs. To induce the fatigue effects the authors subjected the fibers to an uniaxial stress that was ca. 60% of the fracture stress, at an elongation rate of 10 mm/min. Then, to determine the fatigue induced consequences they tested the non-fractured samples under uniaxial stress conditions. It was determined that the residual fatigue strains in the uniaxial tension measurements (subsequent to fatigue) affected most of the mechanical characteristics, such as elastic and hardening moduli, fracture strength and yield strain, of reinforced as well as non-reinforced fibers. The authors concluded that the degradation of mechanical properties as a function of residual strain due to fatigue was greater for the PET/VGCNF composite fibers than for the neat PET fibers [27].

**Figure 5:**
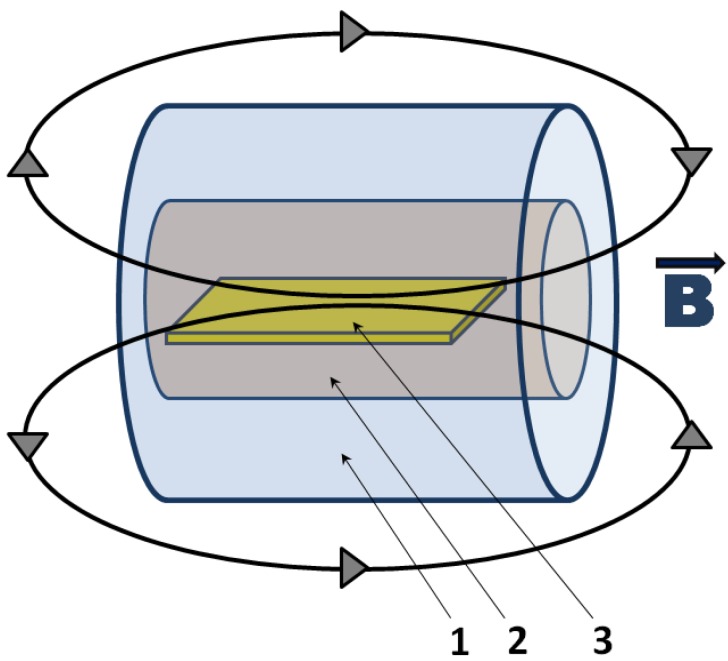
Schematic showing the placement of the samples with respect to the MRI instrument and to the magnetic field (B). The components are: 1 - magnet, 2 - MRI chamber, 3 – PET-SWCNT nanocomposite.

Solution casting is another efficient way to produce PET/CNT composites [28,37]. Wakamatsu *et al.* used a novel approach in which they reacted anionic SWCNTs with a cationic ammonium lipid in several organic solvents (methylene chloride, chloroform, benzene and toluene) [37]. The resultant complex was cast on pretreated transparent PET films to form honeycomb structures. The authors observed that changes in the concentration of SWCNTs, or in the solvent used to prepare the solutions, led to variations in the cell sizes of the honeycomb networks. After removal of the cationic lipid through a simple ion exchange method, the nanotube honeycomb network exhibited thinner skeletons. Additionally, the nanotube network displayed a spectacular change in the resistivity characteristics going from insulating to conducting [37]. Furthermore, Steinert *et al.* prepared a solution blend containing PET and SWCNTs, and cast it onto glass substrates to produce flexible nanocomposite films [28]. During processing the authors used two magnetic resonance imaging (MRI) scanners to produce magnetic fields utilized to induce alignment of the nanotubes in the composites, as indicated in Figure 5. Following the same method they prepared films containing three distinct nanotube loadings (0.5, 1, and 3 wt%). The nanotubes’ degree of alignment could be significantly altered by changing the filler concentration and the strength of the magnetic field, where more anisotropic networks were obtained at elevated SWCNT concentrations (3 wt% vs. 0.5 or 1 wt%) and field strengths (9.4T vs. 3T). The authors concluded that the greatest impact on the conductivity of nanocomposite films was brought mainly by the concentration and degree of dispersion of SWCNTs in the polymer matrix. On the other hand, the alignment of SWCNTs was observed to play a secondary, less important role [28].

Yet another useful approach, besides solution casting, is represented by solution electrospinning [35,38]. Ahn *et al.* used H_2_SO_4_-functionalized MWCNTs dispersed in a PET solution, in trifluoro acetic acid, to prepare nanocomposites via electrospinning [35]. The authors noticed that by increasing the filler concentration they were able to obtain thinner composite nanofibers, and attributed this observation to the shear thinning and charge carrier characteristics of the MWCNTs. Mechanical properties, like tensile strength and modulus, and thermal stability of the resultant nanowebs, as well as the degree of PET crystallinity, were found to increase as a result of an elevated filler loading. Opposite trends were observed for properties like crystallization temperature and elongation at break [35]. Moreover, Chen *et al.* investigated the chain confinement in electrospun PET/MWCNT nanofibers [38]. The authors claim that addition of MWCNTs triggered a more pronounced confinement of PET chains in the electrospun fibers, which in turn led to an increase of the rigid amorphous fraction of the polymer. For example, while in the neat PET fibers the rigid amorphous fraction had a value of 0.23, addition of 2 wt% MWCNTs increased the PET amorphous fraction to 0.64. The authors attributed the decrease in polymer crystallinity to spatial constrains developed during filler addition, which hindered the folding of polymer chains. It was also noticed that while for entirely amorphous composites the filler did not affect the PET chain conformation, for cold crystallized composites the nanotubes favored the development of trans conformers in the electrospun fibers [38].

## 3. Polymer/Metal Nanocomposites

As advanced technologies keep developing every day, there is a constant need for functional materials with special properties and/or combinations of unique properties. Materials based on nano-sized metal particles may help meet many of these needs, and with this thought in mind the central goal of this section is to present and highlight the work recently published on the synthesis and characterization of polymer/metal nanocomposites. In the literature covered here most of the metal-containing materials exhibit excellent mechanical properties and/or exceptional electrical or electro-magnetic characteristics. Additionally, while some of the covered reports emphasize the synthetic approaches taken to obtain metal-based nanocomposites, some others discuss materials properties that allow them to be considered for various applications, such as replacing parts of living tissues. Among the preparative methods presented in the scientific literature only a few are covered in this report. While some sections focus on broader and more general methods, some others present more tailored approaches, targeting either stabilization of the resultant materials, or their magnetic properties, or even other desirable features in the final product.

### 3.1. Polymer/Platinum Nanocomposites

A widely popular matrix for building polymer nanocomposites with Pt is polyaniline (PANI) as exemplified by Liu *et al.*, Nyczyk *et al.*, and Palmero *et al.* [40,98,99]. These papers describe applications containing PANI-Pt nanocomposites used to enhance the catalytic properties of Pt (as verified by catalytic alcohol conversion reactions) by reducing the metal particle size and increasing surface area. Key to the success of the Pt nanocomposites as catalytic systems is a good dispersion of the metal throughout the polymer matrix and a high surface area of the platinum nanoparticles. Nyczyk *et al.* describe polymer nanocomposites of Pt with PANI and its derivatives such as poly(o-methoxyaniline) and poly(o-toluidine) (POT) [40]. The Pt nanoparticles contained in these materials have dimensions between 2 and 4 nm. These are some of the smallest reported Pt particle sizes successfully incorporated into PANI polymer matrices or any of its derivatives. The metal is introduced in the matrices either from an aqueous Pt sol [40], or via electrodeposition [98,99]. A novel composite material based on the electrochemical generation of a layer-by-layer structure of PANI and Pt particles has been prepared by Palmero *et al.* [99]. Additionally, films of PANI synthesized independently by potentiostatic and galvanostatic method, were used by Liu *et al.* as the supporting matrix for loading Pt nanoparticles [98]. The porous network structure of PANI promotes the effective dispersion of Pt particles (about 10–20 nm) and facilitates an unrestricted access of methanol to the catalytic sites, as reported by the same authors [98]. An important aspect in the synthesis and use of these polymer nanocomposites with Pt is prevention from agglomeration of the Pt particles. It is believed that charge transfer interactions between polymer chains and Pt nanoparticles enable the polymer chains to act as stabilizers and to prohibit Pt-nanoparticle agglomeration. Among the polymers studied by Nyczyk *et al.*, POT shows the weakest stabilizing effect [40].

**Figure 6:**
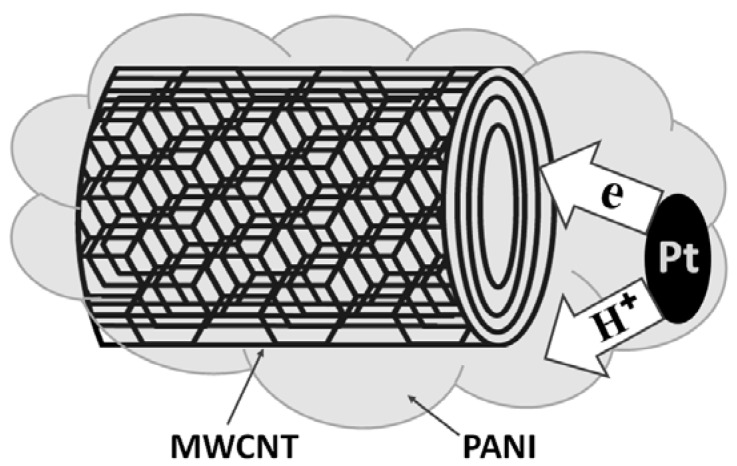
Schematic describing the pathways for the flow of charges in a PANI-Pt nanocomposite deposited on a multiwalled carbon nanotube, as imagined by Sapurina *et al.*

The synthesis of platinum nanoparticles organized inside rod or wire shaped superstructures, with diameters between 1.6–2.3 nm, is described by Tristany *et al.* [100]. Furthermore, carbon nanotubes (CNT) are used as support for polymer nanocomposites with Pt, as described by Sapurina *et al.* and Okamoto *et al.* [101,102]. The great advantage of the carbon nanotubes as support materials is that they facilitate charge transport throughout the assembly, thus greatly increasing the catalytic activity of the polymer nanocomposites with Pt [101]. A schematic showing the increased ability of these systems to distribute charges and their respective pathways is presented in Figure 6. Through their method, Sapurina *et al.* developed novel multipurpose electrode materials of potentially valuable catalytic applications. The materials are composites based on multiwalled carbon nanotubes and electroconducting polymer PANI, organized as “fiber in a jacket”. Nanoparticles of metallic Pt and compounds of transition metals are additionally immobilized in the polymer layer. Experimental results demonstrate that the materials possess high electronic and protonic conductance, thermal stability, hydrophilicity, large specific surface area, and considerable porosity [102]. The polymer nanocomposites with Pt deposited on multiwalled carbon nanotubes may contain various matrixes, such as PANI [102], or poly(benzimidazole) (PBI) [101]. Moreover, nanocomposite materials of PBI incorporating multiwalled carbon nanotubes (MWCNTs) and Pt nanoparticles have been prepared by Okamoto *et al.* [101]. The nanocomposites fabrication involves the preparation of PBI-wrapped MWCNTs (MWCNT/PBI), followed by Pt loading onto the MWCNT/PBI. The average diameter of the Pt particles was found to be 4.8 ± 0.4 nm. Cyclic voltammogram measurements revealed that the composites with Pt nanoparticles deposited on the MWCNT/PBI show higher utilization efficiency (74%) for electrocatalysts than the ones with pristine MWCNT (39%) [101].

Either with the metal supported on CNT, or layered in a conductive polymer-matrix, polymer nanocomposites with small Pt particles show an increased catalytic activity versus materials containing larger Pt particles, as verified by catalytic alcohol conversion reactions [40,98,99,100,101,102,103,104]. Pt can also be used in combination with other metals in nanocomposite-based catalytic systems, such as Fe [104], or Ag [103]. The iron-paired Pt nanocomposites systems exhibit magnetic properties. Magnetic ordered mesoporous carbon with superparamagnetic FePt nanoparticles (average diameters 3–4 nm) has been successfully prepared by Zhu *et al.* via a simple nanocasting route [104]. The magnetic properties are useful in simplifying the separation and regeneration of the catalysts at the end of a process. Furthermore, Compagnini employed two strategies to obtain noble metal particles for nanostructured and polymer-based nanocomposite thin films [103]. On one hand, the reduction of metal-organic compounds, generally used in liquids to obtain metal colloids, has been used directly in the solid state to obtain polymer/metal nanocomposites in a single stage (annealing) after the co-deposition of the two organic species. On the other hand, laser ablation of metallic targets in liquids, has been shown as a new and powerful method to obtain colloids in a wide range of polymer solvents [103].

### 3.2. Polymer/Cobalt Nanocomposites

**Figure 7:**
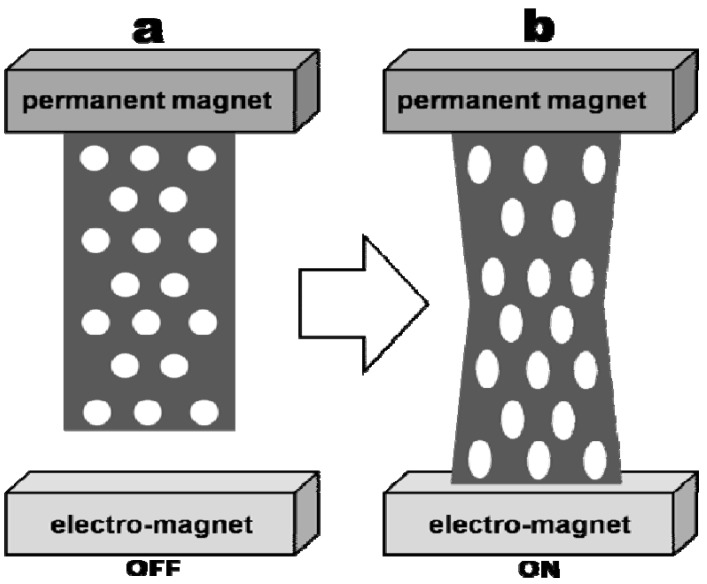
Schematic of a muscle-like Co polymer nanocomposites in a magnetic field.

One of the most interesting applications of Co-polymer nanocomposites materials comes from the biomedical field. Fuhrer *et al.* report the use of a hydrogel as a matrix for incorporation of Co nanoparticles [105]. The mean diameter of Ca particles was found to be ca. 25 nm. The authors observed that the resultant material possesses very high flexibility coupled with surprising strength. They used covalent bonding of the particles to control loss of metal from the nanocomposites due to leaching. As a general desirable feature, it is the authors’ opinion that these materials should contain metals rather than metal oxides. This increases the force to volume ratio for the Co-polymer nanocomposites, making them more suitable for use as magnetic actuators in a muscle-like application [105]. The researchers reported stable hydrogels, loaded up to a value as high as 60 wt%, with good flexibility and shape memory. To increase even further the stability of these systems, Fuhrer *et al.* also reported a *cross-linking of the polymer matrix* approach as a way to reduce Co particle migration. Such a device is represented schematically in Figure 7. The amazing properties of these materials may render them invaluable for use as soft actuators in medical applications [105].

A popular application of biopolymers is related to their ability to control the growth of inorganic particles. Due to differences in the chemical properties and structural variety, biopolymers exhibit various templating efficiencies. Brayner *et al.* used several sources of alginate to control the formation of Co, Ni, and Co/Ni nanoparticles [106]. The authors observed different particle sizes (mostly ranging from 10 nm to 26 nm), structures and magnetic properties in the resulting Co-polymer nanocomposites, all depending on the alginate’s composition. Some of the gels obtained during this work are reported to be stable enough to allow formation of nanoalloys over the entire solid solution domain. The network like structure of the gels is enhanced by the cross-linking effect of metal cations found in close proximity to each other. The gels form cavities that trap the metal and, after reduction, play an active role in controlling the growth of the Co nanoparticles. The anisotropic properties of the cavities reported by the authors can lead to unusual hexagonal compact phase structures in the resulting Co-polymer nanocomposites [106].

Chemical methods for metal deposition represent good choices for creating Co-polymer nanocomposites. One such example was published by Carotenuto *et al.* [20]. The authors describe a method where polymer-embedded Co or Co sulfide clusters are prepared by annealing alkanethiolates of Co dissolved in polymers [20]. Nanoparticles obtained by this approach range in size from 7 to 15 nm. This method proves to be very flexible for obtaining metal polymer nanocomposites, since it allows for the incorporation of various transition metals into polymer matrices. The properties of the resultant materials can be accurately tuned by varying the amount of alkanethiolates introduced and their corresponding annealing times and temperatures [20]. Hybrid materials combining properties of metals, oxides, and polymers can be obtained by mixing these most different materials at the nanometer level, but their success is being strongly dependent on the ability to stabilize and control the interactions at the newly created interfaces [43,107,108]. Owing to such interfacial issues, the incorporation of nanoparticles into engineering thermoplastics by mechanical means (e.g., by melt blending techniques) has always proved to be difficult, especially when it came to the dispersal of inorganic nanoparticles within hydrophobic polymer matrices [109,110,111,112,113,114]. Luechinger *et al.* investigated the possibility to overcome this dispersion problem by mechanically entrapping metal nanoparticles in a grapheme-like carbon shell [115]. The Co core-nanoparticles, which ranged in size from 10 to 50 nm, were covered with a 1–2 nm thick grapheme coating. With this approach the authors aimed at successfully producing metal polymer composites in general, and Co-polymer nanocomposites in particular [115]. One major achievement of the work done by Luechinger *et al.* is the mediation of the surface energy difference between inorganic and organic materials, which was performed by means other than the typical incorporation of chemical additives, such as surfactants. In addition to this advantage, the metal loadings achieved in this study are very high, up to 90%wt, while the resultant materials exhibit an excellent metal dispersion throughout the polymer matrix, all done via a low cost process [115].

Similarly in scope to the research presented by Luechinger *et al.* [115], Pirkkalainen *et al.* [12] investigated the use of a porous cellulose matrix as support for Co nanoparticles, both in amorphous and crystalline form. Making use of a chemical reduction approach, the authors produced Co-polymer nanoparticles within a microcrystalline cellulose matrix [12]. Two kinds of Co containing nanoparticles were successfully obtained: amorphous Co–B or Co oxide composites, prepared through NaBH_4_ reduction and exhibiting poorer ferromagnetic properties, and highly-ordered ferromagnetic hexagonal compact phase (hcp) Co nanocrystals, prepared via a NaH_2_PO_2_ reduction methodology. The authors noticed that the well ordered Co nanoparticles exhibit nanocrystals with average crystallite sizes ranging from 5.3 to 6.3 nm, along with some small amounts of larger micrometric aggregates [12]. Furthermore, Co particles (less than 50 nm diameter) embedded in polytetrafluoroethylene (PTFE) polymer matrix were described by Kwong *et al.* [11]. In their work the research team demonstrated the successful fabrication of Co-PTFE granular composite films by employing a pulse laser deposition (PLD) method [11]. They detected magneto-resistance (MR) values of 4% at room temperature in an applied magnetic field of 8 kOe, and observed that the MR values increase to 6% for measurements performed at a temperature of 20 K [11]. This temperature dependency of the MR was attributed to the spin-dependent tunneling of electrons and the superparamagnetic relaxation behavior of the Co nanoparticles embedded in the PTFE matrix [11]. Magnetic properties of Co polymer nanocomposites were also the subject of the work published by Yurkov *et al.* [44]. The authors present the synthesis of Co nanoparticles with a narrow size distribution (average diameter 8.3 nm), embedded in a polyethylene (PE) matrix. The resultant powder Co-polymer nanocomposite materials were processed in thick films and bulk materials [44]. An increased dielectric permittivity and absorption coefficient is reported by the researchers with the increase in Co nanoparticles concentration, thus leading us to conclude that magnetic properties of the synthesized Co-polymer nanoparticles depend directly on the concentration of metal particles in the composite. Co containing polymer nanocomposites were found to exhibit a higher magnetization (including the remnant one) than that of the iron-containing samples [44].

### 3.3. Polymer/Nickel Nanocomposites

**Figure 8:**
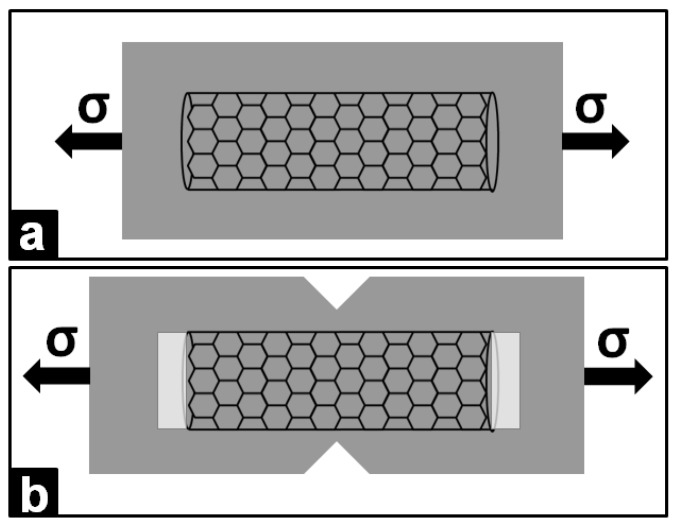
Sketch of a carbon nanotube under tensile pressure (σ), without (a) and with (b) potential separation between the carbon nanotube and matrix. In (b) separation is visible at the two ends of the carbon nanotube, while in (a) the presence of Ni in the polymer matrix prevents the matrix-CNT separation under tensile pressure.

In the work presented by Ghose *et al.*, the goal of the research team was to increase the thermal conductivity of a commercially available ethylene vinyl acetate copolymer (Elvax™ 260) by compounding the copolymer with carbon based nanofillers and including Ni nanostrands (400–800 nm) [116]. The tested material was obtained by extruding the polymer so that the Ni strands were aligned in one direction. A major improvement of thermal conductivity (up to 24 fold) in the direction of alignment was achieved, without any significant loss of flexibility [116]. The authors attributed this improvement in thermal conductivity to a more efficient phonon transfer through the nanotube axis. One interesting application of Ni is represented by its use as a bonding agent in polymer nanocomposites to improve the mechanical properties of the finite material. Such an example is presented in the work by Sun *et al.* [46]. Single-walled carbon nanotubes (SWCNT) are used to reinforce Ni-polymer nanocomposites by bonding the metal to the SWCNT. The authors claim that their method prevents separation of the carbon nanotube from the matrix [46]. An increased strength, up to 3-fold, was noticed in the nanocomposites and the improvement was attributed to the good interfacial bonding between Ni and SWCNT, as well as to the stiffer nature of the matrices [46]. A schematic of the composite material under tensile pressure is shown in Figure 8. Due to the significant differences in the “stretchability” of the CNT and the polymer matrix, separation can occur under load. By loading the polymer matrix with metal nanoparticles, the Young modulus and the stretchability of the matrix is brought closer to that of the CNT, thus reducing the risk of separation under loading [46].

The use of hydrogel networks for growth control of metal nanoparticles is a popular in-situ synthesis technique that can be utilized in conjunction with Ni-based materials [117]. With this technique in mind, Nurettin Sahiner focused his work on preparing metal particles of various sizes inside a three dimensional polymer matrix through the absorption of a NiCl_2_ aqueous solution followed by subsequent reduction to form Ni nanoparticles [117]. The author investigated the impact of cross-linking on the morphology of the hydrogels and the Ni particle size, and found it to have no significant effect on either property. On the other hand, the ion exchange capacity of the matrix material, in this case 2-acrylamido-2-methyl-1-propansulfonic acid (AMPS), was observed to play a major role on the size and loading capacity of the Ni particles [117]. Ni-polymer nanocomposites obtained by this method have potential applications in the biomedical field as catalysts, sensors, and/or signal-triggering delivery systems. The metal particles obtained in this study ranged in size from 40 to 100 nm.

Furthermore, Co and Ni polymer nanocomposites were also the focus of the work introduced by Sarkar *et al.* [45]. The authors present Ni and Co nanoparticles, with sizes between 3 to 5 nm, stable within a polyacrylamide matrix in the presence of air. The investigation of the magnetic properties of these particles showed a low superparamagnetic blocking temperature, as expected for very small Co and Ni crystallites [45]. The work is among the first to present Co-polymer nanocomposites stable in air, at room temperature. Additionally, the advantage of stabilizing magnetic Ni nanoparticles in a gel matrix is underlined in the report [45]. Moreover, Chelebaeva *et al.* present a method of obtaining soluble Ni-polymer nanocomposites by using cyano-ligands [118]. Organic amines were used as stabilizing agents. Particle sizes obtained during this work ranged from 2 to 5 nm, depending on the nature of the metal ion used [118]. According to the authors, these particles were easily precipitated in alcohols and redispersed in organic solvents (ex: hexane). Rather than being the final product, the Ni-polymer nanocomposites served as a precursor component in an interesting work described by Zhao *et al.*, where authors prepared hollow Ni nanospheres from Ni-polymer nanocomposite materials, [119]. The polystyrene (PS) nanospheres used in the study were nickel-plated. Through the use of this approach, the authors were able to obtain uniform Ni particles filled with PS. Subsequently, thermal decomposition of the organic polymer led to formations of the hollow Ni nanospheres, characterized by a high surface area to weight ratio [119]. Concisely, the research team demonstrated an easy, low-cost method of obtaining hollow particles via a Ni-polymer nanocomposite precursor.

On the other hand, focusing on the characterization of nanocomposites rather than synthesis, Mohanraj *et al.* studied the effect of temperature, pressure, and composition on electrical properties of conductive styrene-butadiene rubber-particulate metal alloys [13]. Nanosized particles (smaller than 70 nm) of Cu-Ni alloys were used as fillers, with loading values varied from zero to 40 phr, as reported. While an increase in the temperature was shown to improve the AC conductivity of the Ni-polymer nanocomposites, pressure was observed to result in an exponential decrease in resistivity [13]. It is apparent that the higher the metal loading of the polymer, the better the electrical properties of the nanocomposites are. The improvement can be attributed to the formation of some continuous conductive networks in the rubber matrix [13]. Finally, surface analysis and interfacial effects of filler (Ni) particles on polymer nanocomposite properties were the main goal of the work published by Panda *et al.* [14]. The polymer matrix of choice in this study was polyvinyledene fluoride. The size of the Ni particles, between 20–30 nm, was reported to be directly linked to the enhancement of the low frequency dielectric constant for these materials [14]. This observation has been attributed to the increase in surface area of the smaller size Ni nanoparticles. At the percolation threshold, a higher dielectric constant was observed due to increased leakage current resulting from smaller distances between particles [14].

### 3.4. Polymer/Silver Nanocomposites

Synthesis of hybrid organic/inorganic films by a single step process has been reported by Compton *et al.* [120]. Different complexes (a silver based complex and two palladium based complexes) were used by the authors as precursors in the process of obtaining polymer nanocomposites. Depending on the precursor used and the processing conditions employed, different resulting particle sizes, ranging from 5 to 35 nm, and size distributions were obtained. While a uniform dispersion of nanometer sized spheres was obtained by thermal curing applied to the initial homogeneous (silver complex/polyimide) solution, an increase in the final curing step time at 300 °C was found to lead to a more complex morphology. This morphology change induced small variations in the glass transition temperature (Tg) and the gas transport properties of the Ag-polymer nanocomposite films discussed [120]. In addition to this procedure, a one step photopolymerization process was employed by Balan *et al.* to fabricate Ag-polymer nanocomposites in a poly(ethylene glycol) matrix [47]. In short, the work shows how the radical mechanism that governs the process can be accelerated by the addition of an amine co-initiator, allowing the control of the resultant silver nanoparticles’ sizes (3 to 10 nm). Another approach for the preparation of Ag-polymer nanocomposites, described by Wada *et al.*, involves successive polymerization of silver containing methacrylate monomers [121]. The silver nanoparticles with a mean diameter of 2.8 nm were initially dispersed in the monomer by microwave irradiation, without polymerization. In the authors’ opinion a significant advantage of this process is that the reaction proceeds evenly throughout the reaction vessel, as opposed to thermally driven reactions/processes. The Ag-polymer nanocomposite is later obtained by the ultraviolet (UV) initiated polymerization of the previously metal loaded monomer [121].

**Figure 9:**
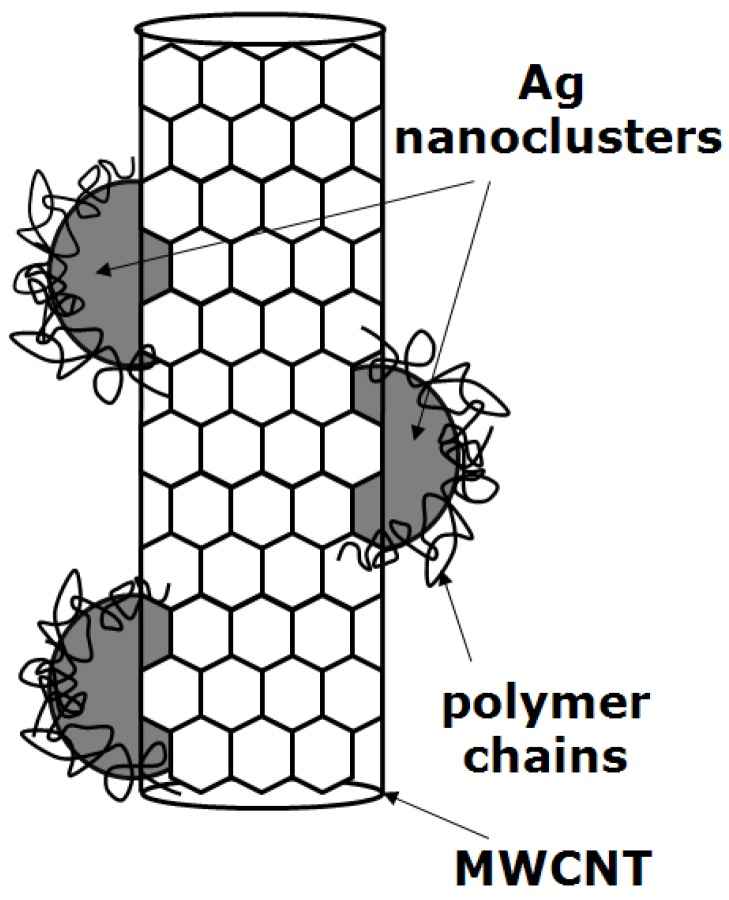
Carbon nanotube/Ag nanohybrids.

An interesting approach to Ag-polymer nanocomposites is depicted in the work published by Gao *et al.* [122]. The process starts with multiwalled carbon nanotubes (MWCNT) which are functionalized in order to become macroinitiators suitable for atom transfer radical polymerization (ATRP) processes. The authors have chosen glycerol monomethacrylate (GMA) as the monomer for the ATRP reaction [122]. Moreover, the researchers used the functionalized MWCNTs to entrap metal ions such as Ag^+^, Co^2+^, Ni^2+^, Au^3+^, La^3+^, and Y^3+^, forming MWCNT-polymer/metal hybrid nanocomposites [122]. The authors reported different shapes, including nanowires and/or necklace-like nanostructures, all depending on the content of the grafted polymer, and the type of the metal loaded. One surprising and unexpected result is the presence of polymer covered-silver nanoclusters, with iameters between 3–10 nm, attached on the convex surface of MWCNTs, as depicted schematically in Figure 9 [122]. Furthermore, the thermal decomposition of (1,1,1,5,5,5-hexafluoroacetylacetonato) silver(I) (AgHFA) in a polymer matrix is the process described by Deshmukh *et al.* to obtain Ag loaded nanocomposite films [123]. In the example described by the authors, AgHFA is decomposed in a poly(methyl methacrylate) film with a silver loading of up to 20 wt%. It is reported that most of the silver is evenly distributed throughout the entire film’s thickness, with only a few percents being situated near the surface. Upon annealing, strong segregation in nanoparticles sizes is observed between the surface, the mid region, and the substrate of these materials. The silver beads have dimensions of 20 to 75 nm towards the surface, 6 nm in the mid region of the films, and 2 to 20 nm at the substrate region [123]. The researchers hope to achieve future control of the conductivity and reflectivity of Ag-polymer nanocomposites films through a rigorous understanding of the phenomenon behind this segregation. In a similar effort, conductivity enhancement in Ag-polymer nanohybrid films was examined by Jiang *et al.* [9]. Starting from silver nanoparticles (average diameter 16 nm) obtained through a chemical vapor condensation method, the authors describe the incorporation of functionalized particles into an epoxy resin (diglycidyl ether of bisphenol A) matrix. Through the use of a diacid surfactant for the functionalization of the silver particles, the researchers were able to obtain polymer nanocomposites with dramatically reduced resistivity. The authors advise that these results were possible due to the sintering of silver nanoparticles, a process that is continued during use, which induces stable properties in the Ag-polymer nanocomposites obtained by this method [9].

Moreover, improvement of the thermal and mechanical properties of poly(vinyl alcohol) (PVA) through silver addition was the goal of the work published by Mbhele *et al.* [124]. Silver was introduced in the polymer matrix by evaporation from water based PVA solutions and silver colloidal mixtures. By loading the polymer matrix with silver nanoparticles, the researchers were able to show an increase in the Tg and thermal stability, and an improvement in the Young’s modulus for the obtained materials [124]. In spite of these promising results, experiments involving high metal loadings revealed a decrease in the stability of the nanocomposites. In consequence, the changes in properties are believed to be proportional with the degrees of metal loading. At last, Mukherjee *et al.* [125] studied the effects of nanoparticles size and density in Ag-polymer nanocomposite thin films. The choice polymer matrix for this study is poly(*o*-methoxyaniline) (POMA). The authors found that the larger particle size silver nanobeads (21.9 ± 1.7 nm) exhibit switching behavior, as shown by the electronic bistability observed in I-V experiments, while the smaller sized particles (8.9 ± 0.8 nm) show rectification properties. They were able to control the electronic properties of the Ag-polymer nanocomposite films by tuning the metal particle size. This choice of polymer helped the authors conclude that the directionality detected in current flow experiments performed on the Ag-polymer composites is due to the asymmetry of the p/n junction assemblies arising from POMA donor (converts to p-type semiconductor) and Ag nanoparticles as acceptor (converts to n-type semiconductor) [125].

### 3.5. Polymer/Palladium Nanocomposites

The catalytic properties of Pd are well known to researchers; however, its inhibitor-like behavior is not so common. Lee *et al.* report both types of behaviors in their Pd-polymer nanocomposites [126]. For example, when combined with polymers like polystyrenes, polypropylene and methacrylates, Pd is observed to retard the thermal decomposition of nanocomposites. This behavior was attributed by the authors to the metal particles hindering the motion of the polymer chains, and thus acting as inhibitors. However, the presence of Pd was found to accelerate the thermal decomposition process of polymers such as polyamide 6 and poly(ethylene terephthalate), effect attributed by the researchers to the catalytic activity exhibited by the Pd nanoparticles. The catalytic properties of Pd are also the main focus of the work presented by Huang *et al.* [42]. A three-step process was used for synthesizing the Pd-polymer nanoparticles. The polymer matrices chosen were polyaniline and polypyrrole. Prior to nanocomposite preparation these matrixes were reduced to their lowest oxidation state in order to obtain a significant uptake of palladium from solution [42]. The catalytic properties of the resultant Pd-containing nanocomposites were investigated by reduction of oxygen dissolved in water, and reduction of nitrobenzene to aniline. The authors present a successful method for obtaining electro-active Pd-containing polymeric nanocomposites, with demonstrated catalytic abilities [42]. Continuing the trend of research underlying the catalytic activity of Pd-polymer nanocomposites materials, Wang *et al.* [127] report a facile way of preparing conductive polymer-supported Pd nanoparticles. The authors used a Pd salt, which was later reduced on the surface of a polyaniline film or membrane, to form a metal-polymer nanocomposite. Cluster sizes observed by the researchers were in the range of 200 to 500 nm, depending on weather a polyaniline membrane or film was used, respectively [127]. It was further demonstrated that those clusters were in fact comprised of much smaller Pd nanoparticles with an average size distribution of 13 nm. The variations in size of the metal particles typically translate into variations in surface area of the Pd particles, and they were further reported to be directly related to the catalytic properties of the resultant compounds [127]. Pd-polymer nanocomposites synthesized via the method described by Wang *et al.* exhibit efficient catalytic properties toward hydrogenation of alkynes and cinnamaldehyde, with good selectivity controlled by a kinetic mechanism. Due to the low cost of materials and ease of synthesis, the authors view such composites as perfect candidates for future development of fuel cell type applications [127].

Two synthetic routes are investigated by Valmikanathan *et al.* [15] in an attempt to obtain Pd-polymer nanocomposite materials: an *in-situ* method and an *ex-situ* method. Both methods are reported to produce materials in which the polymer matrix (polycarbonate) exhibits a glass transition temperature that is 16 °C lower than the one of the pure polymer [15]. One notable difference between the materials resulting from the two methods of synthesis is that the *ex-situ* method yields more dispersed Pd nanoparticles (diameter ca. 15 nm) while the *in-situ* path produces more agglomerated nanoparticles. As a direct result, it is suggested that one should use the *in-situ* synthesis if one wants to obtain electrically conducting Pd-polymer nanocomposites. The *ex-situ* route is more suited for producing a material that was found by the authors [15] to transmit more light than the *in-situ* produced material, for similar metal loadings. Stability of the Pd nanoclusters obtained by either synthetic route in the absence of a capping agent is believed to be a possible issue. On the other hand, the thermal degradation onset temperature of the Pd-polymer nanocomposites materials is shown to be 20 °C to 40 °C higher than the one of the pure polymer matrix [15]. Furthermore, a study on the catalytic activity of Pd nanoparticles encapsulated in spherical polyelectrolyte brushes (SPB) and microgels is presented by Mei *et al.* [128]. The catalytic properties of the resultant Pd-polymer nanocomposites were investigated photometrically, monitoring the reduction of *p*-nitrophenol by NaBH_4_ [128]. In short, the data presented in this work suggests that the activity of the Pd nanospheres (2.4 to 3.8 nm diameter) is highly dependent on the carrier system used, although the authors could not see any clear indication for specific interactions between the polymer chains and the Pd nanoparticles [128].

In the work described by French *et al.*, palladium polymer nanocomposites are used as precursors to Pd-surface-metalized polyimide films [129]. Some of the investigation techniques used to characterize the resultant films include specular and diffuse reflectivity and conductivity measurements as a function of curing time and temperature. The authors observed that during the curing step, Pd in the 2+ oxidation state undergoes reduction to Pd metal [129]. At around 250 °C very small particles (ca. 3 to 10 nm) of Pd metal are formed at the surface, and they increase in size and agglomeration with increasing curing temperatures. Based on these observations, the researchers speculate that only a relatively narrow processing window exists during the thermal curing protocol where the optimum film, with regard to specular reflectivity, conductivity, and mechanical integrity, can be produced [129]. Because Pd is a very versatile catalyst, utilized in synthesis of various chemical compounds, including metal-polymer nanocomposites, Krebs *et al.* [130] focused their studies on the effects of the remnant Pd catalyst particles in polymer nanocomposites. The authors chose two synthetic routes for obtaining electrically conductive, thin polymer films. While one route required the use of Pd catalyst, the second route was based on a condensation reaction, with no need for Pd [130]. The authors showed that palladium nanoparticles (20 nm diameter) can act as very efficient charge carriers, influencing the properties of thin polymer films. The inability to completely remove all Pd catalyst traces severely impairs the electrical properties of the thin polymer films, limiting their application for electroactive devices, for which reason caution is advised when Pd is used as a catalyst in the polymerization processes (particularly when electrically active materials are sought) [130].

### 3.6. Polymer/Gold Nanocomposites

**Figure 10:**
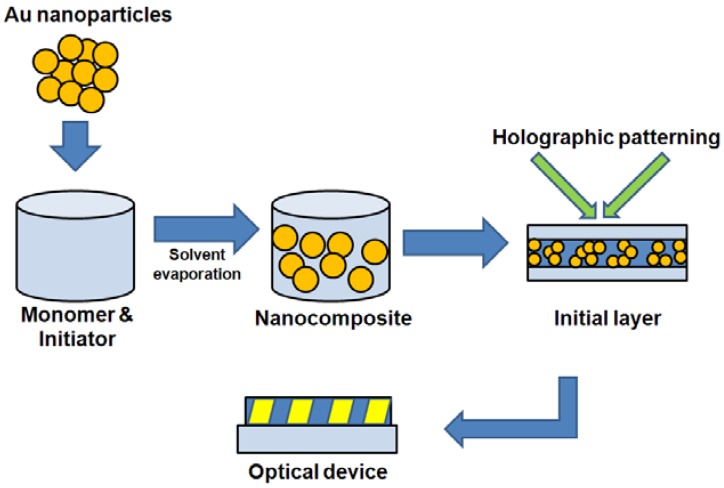
Schematic of the nanocomposite preparation and optical active element fabrication.

The preparation of regular and mechanically robust electric components based on Au-(conducting polymer) nanostructured materials is described by Zotti *et al.* [41]. The authors produced regular films, full monolayers and multilayers via solution processing, through the use of gold nanoparticles (average diameter 5 nm) and soluble conductive polymers, such as polypyrrole or poly(3,4-ethylenedioxy-thiophene). The resulting materials were reported to have good conductivities, in the order of 3–6 × 10^-2^ Scm^-1^ [41]. In addition, the conductive polymers used in the study were found to strongly act as aggregating agents for the gold nanoparticles. The fact that addition of polyconjugated electron linkers did not modify the electron transport led the authors to conclude that the electron transport properties of the Au-polymer nanocomposites described in this work are mainly a result of gold-polymer contacts [41]. Furthermore, two acrylate monomers with different functionalities and gold nanoparticles were the starting materials for the Au-polymer nanocomposites synthesized by Goldberg *et al.* [131]. The authors utilized an original synthetic approach that includes a holographic photopolymerization methodology, schematically represented in Figure 10. To ensure the stability and dispersbility of the Au particles (1.5–3 nm core diameter) in the acrylic monomers, the particles were coated with an ester terminated alkanethiol [131]. The researchers showed that increasing the Au loading of the polymer matrix decreases significantly the rate of polymerization. Their preliminary investigations proved that the polymer-Au nanoparticle gratings (periodically ordered metal nanostructures) exhibit nonlinear optical properties [131].

Moreover, Hata *et al.* proved that the intercalation of a basic polyelectrolyte poly(allylamine) (PAA) into a synthetic Na-fluoromica produces Au-polymer nanocomposites with very different properties under different pH conditions [132]. At high pH values the saturation loading was found to be lower, but the free energy of intercalation was observed to be more favorable. At low pH, the polymer chains are completely protonated, and at high loadings, chloride ions (from the addition of HAuCl_4_) are also intercalated to screen the repulsive interactions between adjacent polycation chains [132]. The optimum pH for intercalating Au nanoparticles (smaller than 10 nm diameter) was reported to be 11.0 where typically the matrix has relatively little charge. At a high loading of Au nanoparticles, the authors observed some unusual features in the long-wavelength region (570–730 nm) of the plasmon resonance spectrum, features reportedly arising from interlayer particle interactions [132]. The work illustrates a convenient route for obtaining high-quality dispersions of noble metal nanoparticles intercalated into layered materials, that offers a rather attractive potential for new optical applications based on plasmon resonance. Li *et al.* obtained novel nanocomposites of gold nanoparticles and poly(4-vinylpyridine) by using surface initiated atom-transfer radical polymerization (ATRP) at ambient conditions [133]. The Au nanoparticles, with an average diameter of 20 nm, were stabilized using citrates, and then modified with a disulfide initiator in order to be active in the ATRP initiation. Authors claim that the polymerization reaction takes place at the surface of the gold particles and that due to the presence of protonated pyridine groups, the Au-polymer nanocomposite materials are pH responsive. The polymer chains attached to the gold nanoparticles are expanded at low pH and collapsed at high pH values [133]. The researchers suggests that materials exhibiting this type of behavior can act as smart catalysts [133].

Furthermore, Uhlenhaut *et al.* were able to obtain nanocomposites of poly(dimethylsiloxane) (PDMS) and thiol-modified gold particles (2–3 nm average diameter) that have the ability of offering a versatile reversible-color system, as a result of kinetically controlled particle dispersion states [134]. The authors claim that due to the elastic properties of the polymer matrix, dichroic states can be generated and can be transformed again to optically isotropic states by swelling. However, upon heating to 220–240 °C for a few minutes, irreversible thermochromic effects were detected as a result of desorption of alkanethiol molecules from the gold surfaces, which leads to the irreversible particle aggregate formation [134]. In addition to this work, several methods for synthesizing arrays of Au-conductive polymer nanocomposites and solid conductive polymer nanoparticles were studied by Marinakos *et al.* [135]. Although the authors believe that such Au-conductive polymer nanocomposite materials are ideally suited for studying electron hopping in nanoscale solid-state systems, the main goal of their research was the use of metal nanoparticles as templates for forming hollow polymeric nanocapsules [135]. It appears that the possibility of using Au-thiolate chemistry to introduce a desired molecule into the interior capsule core was the driving force in the study [135]. For this work the authors employed suspensions of citrate-stabilized Au nanoparticles with diameters of either 8 nm or 30 nm.

Moreover, Park *et al.* reported on the preparation of blue polymer light-emitting diodes (PLED) with improved luminescent stability [136]. The PLEDs were obtained by incorporating 5–10 nm gold nanoparticles as the quenchers of the triplet states of blue emitting polymer. The presence of the Au nanoparticles conferred several advantages to these devices: for example the gold-doped PLEDs are protected from oxidation by the quenching of the triplet state of the polymer. Additionally, the authors noticed that the Au nanoparticles appear to modify the interfacial morphology of the nanocomposites in manner that facilitates electron injection while blocking hole migration [136]. In short, the materials doped with Au nanoparticles showed greater external quantum efficiencies than the pristine PLEDs. Moreover, a polythiophene-gold nanoparticle alternate network film was prepared by Tanaka *et al.* [137]. The described synthesis involves mixing a thiol group-terminated polythiophene and gold nanoparticles (diameter 3.3 ± 0.8 nm) that result in nanocomposite materials exhibiting excellent conductive characteristics, and relatively high electric conductivity. These properties coupled with an extremely low activation energy led the researchers to conclude that the intrinsic conductive characteristic of the polythiophene backbone can be evaluated by connecting the polythiophene with Au nanoparticles through the linkage of conjugated S-Au bonds [137].

Gold-fluoropolymer nanocomposites can be utilized as vapor sensing devices, as indicated by Cioffi *et al.* who studied the composition and structure of such materials [138]. The purpose of their work was to better understand the swelling mechanism behind the material’s vapor sensing capability. Although transmission electron microscopy results showed a uniform horizontal distribution of the Au nanoparticles (diameters between 2–8 nm) in the polymer layer, when analyzed by angle resolved X-ray photoelectron spectroscopy, a vertical concentration gradient of the Au nanoparticles was identified [138]. It was also proven that the extent of cross-linking in the polymer matrix increases with the gold particle concentration, and that the interface between the metal and polymer is comprised mostly from Au oxides and fluorides. The authors expect the Au oxides and fluorides species to play a critical role in the gas swelling phenomenon that occurs during the exposure of the materials to polar solvents [138]. At last, Laicer *et al.* studied the mechanism of morphological seeding in block-copolymer nanocomposites [139]. In their work the authors combined cylinder phases of polystyrene-block-polyisoprene diblock (as a solution in dibutylphthalate) and poly(styrene-blockisoprene-block-styrene) triblock (as a blend with homopolystyrene) copolymers with gold nanorods of different diameters (30, 100, 180 and 400 nm) and surface treatments. The overall goal of the work is to illustrate a general mechanism for the kinetic templating of block-copolymer domains from Au nanoparticle seeds. The researchers suggest that both kinetic and thermodynamic factors need to be considered when looking at the design rules for templating nanocomposite block copolymers [139]. It is also stated that it is possible to actively orient the block copolymers via kinetic templating throughout the bulk composite if electric, magnetic, or shear fields are applied to align nanorods suspended in an isotropic polymer melt prior to domain nucleation [139].

### 3.7. Other Metals/Polymer Nanocomposites

Iron-containing nanocomposites represent an important class of materials. A simple approach for fabricating a robust vinyl ester resin nanocomposite, reinforced with iron nanoparticles, is demonstrated by Guo *et al.* [140]. This approach eliminates the need for any surfactant or coupling agent. The authors show that the resin is chemically bound onto the nanoparticle surface and protects the iron nanoparticles (average diameter 20 nm) from agglomeration and oxidation. The mechanical properties of the resultant material are enhanced, as proven by tensile strength and Young’s modulus values larger than those of cured pure resin. The resulting Fe-polymer nanocomposite material is also reported to be magnetically harder, with an increased thermal stability, showing ferromagnetic properties at room temperature [140]. The authors believe that these materials have potential applications in the marine systems, magnetoresistive sensors, and microwave absorption systems [140]. In addition, Baker *et al.* used iron along with its oxides to produce Fe-oxide/Fe core shell nanoparticles (average size 13 ± 2 nm) dispersed in polymethylmethacrylate (PMMA) films in varying concentrations [141]. The authors observed that the magnetic properties of the composite materials depend on the amount of Fe-oxide coating the iron particles, and hypothesized that this behavior is a result of a heightened anisotropy barrier that results from the ferrimagnetic oxide shell and the exchange interaction with the Fe core. Reduced dipolar interactions between particles are reported, which are believed to be triggered by the particles being separated from each other. As a result, the researchers observed an increase in coercivity, and a slow relaxation rate in thermoremanent magnetization experiments [141]. The optical properties of iron-polymer nanocomposites were studied by Ushakov *et al.* [142]. The polymer matrix chosen for the work was a linear low density polyethylene. Different iron concentrations in the polyethylene matrix were investigated at room temperature, in the visible and near-infrared regions [142]. The authors demonstrated that optical methods, such as linear optical spectroscopy, facilitate the size determination of ultra-small nanoparticles (through direct energy measurements), determination which is reportedly rather difficult to perform by other known methods [142]. The metal particles examined in this study had diameters between 2–8 nm.

Another important class of metal-containing materials is represented by the polymer/aluminum nanocomposites. Huang *et al.* focused their efforts on studying polyethylene-aluminum (PE/Al) nanocomposites [143,144,145]. In their initial study published in 2008 the authors describe the preparation of Al-polymer nanocomposites with concentrations ranging from 1 wt% to 48 wt% metal dispersed in a PE matrix, employing Al particles with diameters of 102 nm [143]. Nanoscale dispersions were observed at concentrations lower than 4 wt%, and agglomerates were detected to be present at higher concentrations. The nanocomposites obtained in this work exhibited electrical and mechanical properties believed to be good enough for practical applications [143]. In an attempt to investigate the dispersion of surface treated Al nanoparticles in the polymer matrix, the same group later prepared PE/Al nanocomposites, via solution compounding, using Al nanoparticles (100 nm diameter) with and without surface modification respectively [145]. The authors observed a strong correlation between the time and concentration dependences of DC-conductivity and the rheological properties of different nanocomposite systems. It was also noticed that the modification of the Al nanoparticles with a silane coupling agent could lead to improved dispersion of the Al particles in the polymer matrix [145]. Conclusions drawn by the researchers illustrate two mechanisms that affect the rheological properties of the Al/PE nanocomposites: while the particle–particle interaction is the dominant mechanism in the “non-surface-treated” composites, the particle-polymer interaction is the dominant one in the “surface-treated” counterparts [145]. One year later, another study from the same group focused on the effects of surface treatment of Al nanoparticles on the electrical properties of linear low density polyethylene composites [144]. It was found that, due to the surface treatment, the Al nanoparticles with non-polar octyl groups not only increased the percolation threshold and the resistivity, but also improved the dielectric properties as compared to the composites filled with non-treated Al [144]. The researchers also noticed that the surface allows to easily control the frequency and concentration dependences of dielectric constant and provided an excellent approach to considerably reduce the dielectric loss of the nanocomposites. The authors consider the findings to be very encouraging for practical applications of the polymer/Al nanocomposites in the electrical and electronic industries [143,144,145]. In conclusion, the improved electrical properties could be directly attributed to the good dispersion and special electrical features of the surface-treated nanoparticles in the polymer matrix [144].

Finally, the last important class of metal-containing nanocomposites covered in this report is the group of copper (Cu)-based materials. A novel in-situ synthetic method for the preparation of copper-polymer nanocomposite materials is presented by Mallick *et al.* [146]. The authors used o-toluidine and cupric sulfate as precursor materials. While during the chemical reaction o-toluidine was oxidized to form poly(*o*-toluidine), the cupric sulfate was reduced to form copper nanoparticles with diameters of ca. 2.5 nm [146]. This procedure demonstrates a single step synthetic route for the preparation of Al–polymer nanocomposite materials. The conductivity of the poly-(*o*-toluidine)-Cu composite material was found to be approximately 10^-3^ S cm^-1^. As future work, the authors aim to further investigate the potential applications and the larger scale production of such composites [146]. Moreover, the recent availability of nanomaterials suitable to make conductive composites using much smaller particles than in the past served as a driving force for the study performed by Untereker *et al.* [147]. The researchers focused on answering two questions in their study: how does particle size influence the maximum conductivity, and what is the maximum achievable conductivity for these materials. The authors did not find great variations in the conductivity of materials like platinum, carbon black, and silver particles dispersed in polyurethane composites, particles with sizes from micrometer to nanometer range [147]. A surprising finding was that in all the examples studied, the highest conductivity achieved was only about 1% of that of the pure bulk conductive materials. Further experiments to emulate these conductive composites with platinum, carbon black, copper, and nickel particles, and without polymer matrix, showed similar results, leading to the conclusion that the composite systems are intrinsically limited by the contact between filler particles [147]. This finding triggered the hypothesis that small sized particles alone do not improve the making of conductive composite materials. The authors believe, however, that high conductivities might be accomplished if the filler contact density is reduced by sintering or by using high-aspect ratio fillers [147]. At last, a polymer based nanocomposite containing stabilized copper nanoparticles was proposed to serve as a biostatic coating by Cioffi *et al.* [148]. The authors investigated correlations between biological effects and material properties and showed a slow release of metal species to act as growth inhibitors for living organisms [148]. The center goal of the study was to evaluate metal-polymer nanocomposite materials for perspective applications in the preparation of antibacterial paints/coatings to be used in household, biomedical/ hospital, and aerospace industries [148]. The Cu nanoparticles described in this study were found to have diameters around 4.6–4.9 nm.

## 4. Polymer/Clay Nanocomposites

The exfoliation, intercalation and aggregation of clays in PEO nanocomposites have been extensively studied in the past. Although in complicated multi-component systems it is difficult to draw conclusions if too many parameters are varied, the reality is that the functionality of each component is often altered by addition of another. Due to this fact and for a more complete picture of how a system behaves in a real environment, all the constituents must be considered at once. Typically, to examine the polymer-clay interactions a combination of methods is advisable. Among them, microscopy and scattering are techniques for studying structure and providing a measure of size, shape and interfacial polymer conformation and are frequently employed by various researchers. Additionally, rheology and mechanical testing are much utilized techniques because they can adequately distinguish between properties of chemically versus physically cross-linked polymer-clay materials. In this chapter are reviewed and discussed only the polymer nanocomposites based on Laponite^®^ and montmorillonite, since these two clays are among the most studied and researched.

### 4.1. Poly(Urethane Urea) Composites

**Figure 11:**
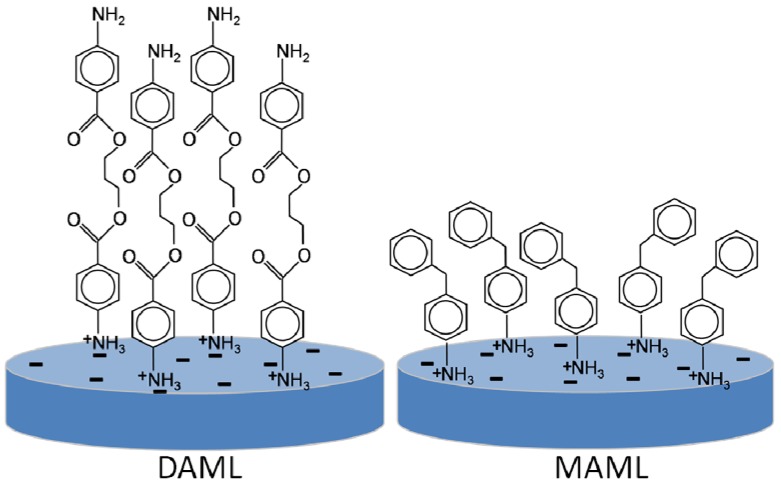
Schematic illustrating the chemical structure of modified Laponite^®^s. For simplicity, only one side of modification is presented.

Poly(urethane ureas) (PUU) are a class of copolymers that exhibit many engineering applications owing to their dual morphology. The presence of both hard and soft segments in the polymer structure contributes to the stiffness, strength and elastic nature of the polyurethane. Despite the excellent elastic properties, the utilization of PUU is limited by the melting of the hard segments at elevated temperatures. One way to improve the mechanical and thermal properties of PUU is the dispersion of inorganic particles into the polymer matrix. In their work, Sormana *et al.* reported the preparation of PUU nanocomposites filled with monoamine-modified Laponite^®^ (MAML) or diamine-modified Laponite^®^ (DAML) [149]. A schematic showing the chemical structure of modified Laponite^®^s is presented in Figure 11. An ion exchange technique was used for the coating on the surface of the Laponite^®^ clay with the organic diamine or monoamine. The authors observed that both the tensile strength and the elongation at break of the polyurethane-clay nanocomposites (PUCN) containing DAML increased when compared with the neat PUU. This behavior was the result of a better interaction of the inorganic phase with the hard segments of the PUU. DSC thermograms exhibited nearly indistinguishable differences in the glass transition temperatures of the PUCN and pure PUU, suggesting that the modified Laponite^®^ did not interfere with the soft segment phase-free volume of the PUU. The nanocomposites containing DAML revealed higher tensile strength than those with MAML. Mishra *et al.* utilized the same technique for the modification of Laponite^®^ clay with dodecylamine hydrochloride [150]. The PUCN (synthesized by *in situ* polymerization technique) showed an increase in the thermal stability; however, their elongation at break was decreased due to the preference of modified Laponite^®^ for the more polar and soft propylene glycol-segments of the PUU. Surprisingly, when the PUCN were prepared by a solution mixing technique, Mishra *et al.* observed that the modified Laponite^®^ had affinity for the hard segments of the PUU [151]. The resulting PUCN showed an increase in the storage modulus at 140 °C.

On the other hand, Korley *et al.* used a different approach to improve the thermomechanical properties of the PUCN [152]. A solvent exchange process was employed to disperse neat Laponite^®^ clay in PUU polymers containing a hydrophobic hard segment, 1,6-hexamethylene diisocyanate – 1,4-butanediol (HDI-BDO), and a soft segment that was either a hydrophilic poly(ethylene oxide)-poly(propylene oxide)-poly(ethylene oxide) (PEO-PPO-PEO) domain, or a hydrophobic poly(tetra-methylene oxide) (PTMO) one. The loaded PEO-PPO-PEO-based PUU nanocomposites showed a decrease in strength and toughness when compared with the same unloaded systems or with the PTMO-based PUU loaded nanocomposites. Dynamic mechanical analysis (DMA) and differential scanning calorimetry (DSC) tests revealed that upon loading with Laponite^®^ clay the mobility of the soft segments in the PEO-PPO-PEO-based PUU composites was reduced, suggesting a favored attraction of the clay platelets to the polar soft segments that negatively affected the mechanical properties of the material. In contrast, the Laponite^®^ platelets in the PTMO-based PUU nanocomposites were primarily confined to the hard domains enhancing the toughness and the strength while maintaining the flexibility of the materials.

### 4.2. Poly(Methyl Methacrylate)/Laponite^®^ Composites

Poly(methyl methacrylate) (PMMA) is a thermoplastic polymer widely used due to its low cost and facile handling and processing. However, when subjected to stress the PMMA is predisposed to fracture. The dispersion of Laponite^®^ clay platelets into the polymer matrix is employed as a way to improve the properties of PMMA. Fang *et al.* used a Pickering emulsion (an emulsion system stabilized by particles) polymerization to synthesize Laponite^®^-mediated poly (methyl methacrylate) (PMMA) nanospheres [153]. The authors prepared the emulsion by mixing a solution of Laponite^®^ in distilled water with a solution of methylmetacrylate in *n-*heptane, where the Laponite^®^ clay was used as a stabilizer. The polymerization of MMA was initiated by 2,2’-azobis(2-methylpropionamide) dihydrochloride. The resultant PMMA nanoparticles, wrapped in Laponite^®^ clay, showed superior electrical conductivity when compared to neat PMMA. The authors hypothesized that the increase in the electrical conductivity of the nanospheres is caused by the intrinsic electrical properties of Laponite^®^ clay.

**Figure 12:**
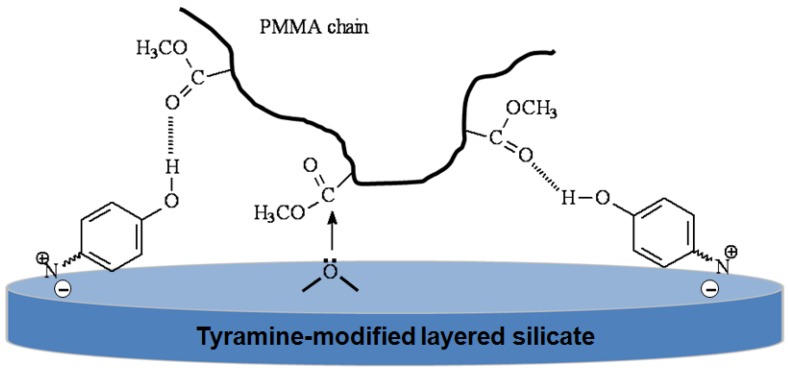
Schematic showing interaction and hydrogen bonding between modified clay and PMMA.

Chang *et al.* used tyramine-modified Laponite^®^ clay to enhance blending with the PMMA chains [154]. Tyramine hydrochloride was used for the modification of Laponite^®^ clay. Due to the presence of silicate-oxygens and of the phenol groups partially covering the silicate layers, the PMMA macromolecules were absorbed onto the silicate through hydrogen bonding and the resultant nanocomposites displayed exfoliated and intercalated morphologies of the modified Laponite^®^ into the PMMA matrix. A schematic of the tyramine-modified layered Laponite^®^ clay is depicted in Figure 12. The authors concluded that the size of the modified clay in the polymer matrix critically impacts the structure and thermal properties of the nanocomposites. For example, when employing silicate clays with a larger diameter, the corresponding PMMA matrix consists of intercalated nanocomposites. An explanation for this behavior is that during the evaporation of the solvent the silicate layers with larger diameter tend to accumulate resulting in silicate-rich domains. Wang *et al.* employed a different technique to enhance blending and mechanical properties of the PMMA nanocomposites by chemically bonding dual-functionalized Laponite^®^ clay with PMMA [155]. The dual- functionalized Laponite^®^ clay was synthesized via edge functionalization (silanol condensation) of the Laponite^®^ with γ-methacryloxypropyltrimethoxysilane (MPTS) followed by cation exchange reactions with cetyltrimethylammonium bromide (CTAB). The dual-functionalized Laponite^®^ was then copolymerized with MMA to develop a polymer-grafted PMMA nanocomposite through a free radical polymerization. The silanol condensation was performed to avoid the linkage of the clay sheets, while the CTAB intercalation allowed the polymer to break through the clay sheets to a greater extent. The DMA results showed that the PMMA-Laponite^®^-MPTS-CTAB nanocomposites have a higher storage modulus than PMMA and PMMA-Laponite^®^-MPTS nanocomposites. Finally, Wheeler *et al.* used the same technique for the preparation of PMMA nanocomposites only that the Laponite^®^ clay was covalently bonded with a different acrylate/methacrylate compound and the binding of the polymer was performed via two types of polymerization: 1) *in situ* polymerization of MMA using AIBN as a free radical initiator; 2) brush polymerization using atom transfer radical polymerization (ATRP) initiator on Laponite^®^ clay [156]. The TEM analysis indicates that the CTAB treated nanocomposites clays show some separated sheets but aggregates are predominant; these results are in contradiction with those obtained by Wang *et al.* [155].

### 4.3. Polystyrene/Laponite^®^ Composites

Konn *et al.* used a nitroxide-mediated polymerization (NMP) of styrene-modified Laponite^®^ clay to control the growth of polystyrene (PS) chains from the surface of the silicate clay, as a way to enhance dispersability of Laponite^®^ platelets in polar and nonpolar monomers [157]. The Laponite^®^ clay was modified by performing cation exchange with a quaternary ammonium alkoxyamine that had incorporated in its structure a *N*-tert-butyl-*N*-[1-diethylphosphono-(2,2-dimethylpropyl)] (DEPN) group. The quaternary ammonium functional group is responsible for the attachment to the Laponite^®^ clay while the DEPN is used as an initiator for the NMP technique. The authors observed that when low molecular weights of PS-functionalized Laponite^®^ platelets were dispersed in tetrahydrofuran (THF), the corresponding clay suspension also included some clay aggregates. After increasing the polymer chain length the clay dispersion was promoted as a result of steric barrier created by polymer chains surrounding the clay platelets. Dynamic light scattering (DLS) measurements of the suspensions showed that PS-functionalized Laponite^®^ exhibited a good dispersability and stability in organic, polar, and nonpolar monomers. X-ray diffraction (XRD) measurements of films obtained from these suspensions revealed that even after casting the clay platelets were still fully exfoliated.

Furthermore, Herrera *et al.* used seeded emulsion (co)polymerization to synthesize polymer/modified-Laponite^®^ composite colloids [158]. The Laponite^®^ clay was functionalized with a free radical initiator, 2,2’-azobis(2-methylpropionamide) dihydrochloride, by cation exchange. Because the corresponding clay platelets were strongly aggregated in the presence of water, tetrasodium pyrophosphate (a peptizing agent) was used to accomplish a good dispersion of the clay in water. Next, the emulsion (co)polymerization was accomplished using styrene and butyl acrylate. The authors observed that increasing the amount of initiator used for the modification of Laponite^®^ clay resulted in silicate aggregates with larger sizes and composite particles with improved morphologies. In a later work, Herrera *et al.* used the earlier mentioned colloids to prepare films by water evaporation and composite particles coalescence [159]. The resultant transparent films (containing 10wt% modified-Laponite^®^) exhibited an increase in the thermal stability and glass transition of 18 °C and 12 °C, respectively when compared to the properties of the copolymer in the absence of modified-Laponite^®^ platelets. Ruggerone *et al.* also employed an emulsion polymerization technique to synthesize polystyrene-modified-Laponite^®^ nanocomposites [160]. The Laponite^®^ clay was modified with 5 wt% macromonomer (poly(ethylene oxide)1000 monomethyl ether metacrylate) while the polymerization was performed with styrene using 2,2` azobis(cyanopentanoic acid) as initiator. The authors noticed that the extent of the modified-Laponite^®^ significantly affected the mechanical properties of the PS-Laponite^®^ nanocomposites films. For example, films with a loading of 5wt% modified-Laponite^®^ displayed an increase in the stiffness and tensile strength when compared with the unmodified films. On the other hand, films with a content of 10 and 20 wt% modified-Laponite^®^ revealed reduced mechanical properties. This behavior was explained by the presence of large silicate clay agglomerates formed at high clay filling.

At last, Caruso *et al.* employed a layer-by-layer self-assembly technique for the coating of PS spheres templates with Laponite^®^ clay multilayers [161]. Prior to coating with silicate clay, the PS particles were modified by the adsorption of 3 layers of positively and negatively charged polyelectrolytes [poly(diallyldimethylammonium chloride) and poly(sodium 4-styrenesulfonate)]. The benefit of this procedure was that the thickness of the multilayer wall and the diameter of the composite particles could be controlled by adjusting the initial PS diameter and the number of layers deposited on the templates. The authors demonstrated that through the use of this technique, the Laponite^®^ clay is a potential candidate for building blocks utilized in hollow sphere fabrication. These Laponite^®^ spheres were obtained by heating the PS colloidal templates at elevated temperatures for the removal of the organic core.

### 4.4. Poly(Ethylene Oxide)/Laponite^®^ Composites

Loizou *et al.* investigated the dynamic responses in poly(ethylene oxide) (PEO)/Laponite^®^ nanocomposite hydrogels by means of SANS and rheology measurements [2]. The authors employed different molecular weight PEOs for the preparation of gels with the same mass composition (3% Laponite^®^ and 2% PEO). All samples had a pH of 10 and a similar NaCl concentration. SANS measurements indicated that at rest no anisotropy was present for any of the systems while in the presence of an applied flow field, anisotropy developed into the gels containing the higher molecular weights of PEO. It was also observed that the anisotropy increases with the increasing molecular weight of polymer. This behavior was explained by an enhancement of polymer-Laponite^®^ clay interconnections as a result of the increase of the polymer chain length. The rheological measurements conducted on the gels containing the higher molecular weight PEOs showed a frequency-dependent storage modulus suggesting the formation of a transient network between the Laponite^®^ clay and the polymer. A network-like structure on the micron and nanometer length scale was confirmed by SEM and TEM images of the hydrogels [1]. The complexation of PEO oxygens with the Na^+^ counterions of the silicate clay was also confirmed by solid-state NMR experiments which revealed a decrease in the segmental mobility of the PEO [162]. Stefanescu *et al.* studied the structure and the thermal properties of multilayered Laponite^®^/PEO nanocomposites prepared by shear-spreading the PEO/Laponite^®^ nanocomposite hydrogels [54]. SEM and SANS measurements obtained from the films indicated that the Laponite^®^ platelets oriented in the direction of spreading, resulting in well dispersed and oriented multilayers. XRD data showed an increase in the order reflection for the films with higher amounts of Laponite^®^ due to a dense packing of the platelets that had a tendency to stack and orient. The authors also observed that different compositions and different polymer molecular weight affected the crystallinity of the nanocomposite films. As indicated by DSC data, the crystallinity of the films increased with the increase of the PEO molecular weight while lower melting temperatures were observed with the increase of Laponite^®^ concentration. Later on, hybrid polymer-clay nanocomposites were investigated by Stefanescu *et al.* [6]. The authors prepared nanocomposite films containing two types of clay (Laponite^®^ and montmorillonite) as a way to improve the properties of the films. DSC measurements revealed that higher amounts of montmorillonite in the system led to an increase of crystallinity of PEO in the nanocomposite. This behavior was the result of a much lower surface per gram of montmorillonite available for PEO coordination than Laponite^®^. As a consequence the uncoordinated PEO chains tended to move and rearrange increasing in this way the crystallinity of the films. However, the XRD showed that higher montmorillonite amounts in the nanocomposite films improved the orientation of the clay platelets. The increased size of the montmorillonite clay was responsible for the behavior where the clay platelets followed the axis induced by the spreading direction as opposed to the Laponite^®^ clay that gradually lost parallelism due to their lack of high aspect-ratio. Stefanescu *et al.* also performed a mechanical study on the hybrid PEO-clay nanocomposite gels and multilayered films [55]. Rheological measurements of the hybrid nanocomposite gels showed a shear thinning behavior which indicated an overall orientation of the nanostructures in the gels. Moreover, the storage and loss modulus increased with the increasing amount of Laponite^®^ into the sample due to improved distribution of surface area of the clay which elevated the viscosity of Laponite^®^ rich gels. However, DMA measurements of the solid multilayered films revealed that the behavior of elastic and loss modulus was opposite to the one observed for the storage and loss modulus of the precursor nanocomposite gels. The increase in the crystalline fraction of the PEO in the films containing higher amounts of montmorillonite was found responsible for this behavior.

Loyens *et al.* prepared PEO/Laponite^®^ and PEO/modified Laponite^®^ nanocomposites via a melt-compounding procedure [163]. The modification of Laponite^®^ with poly(ethylene glycol) components was performed in order to increase the compatibility of the clay with PEO. The authors observed that the addition of Laponite^®^ clay inhibited the crystallization of PEO. This behavior was the result of the coordination of PEO with the Laponite^®^ platelets which restricted the mobility of the polymer chains. TGA measurements indicated that nanocomposites based on modified Laponite^®^ exhibited an increased thermal stability at low clay loadings while the nanocomposites based on pure Laponite^®^ showed an increase in thermal stability only at high clay concentrations. Only a slight improvement of the mechanical properties was observed for the PEO nanocomposites based on pure or modified Laponite^®^. In a later work, Loyens *et al.* demonstrated that the presence of a salt (NaClO_4_) in melt-compounded PEO/Laponite^®^ nanocomposites did not exhibit improved mechanical properties nor increased ionic conductivity when compared to the corresponding PEO/ NaClO_4_ complex [52]. However, when the Laponite^®^ clay was replaced with Cloisite30B the nanocomposites showed excellent mechanical properties and good ionic conductivity.

### 4.5. Diblock and Triblock Copolymers/Laponite^®^ Composites

**Figure 13:**
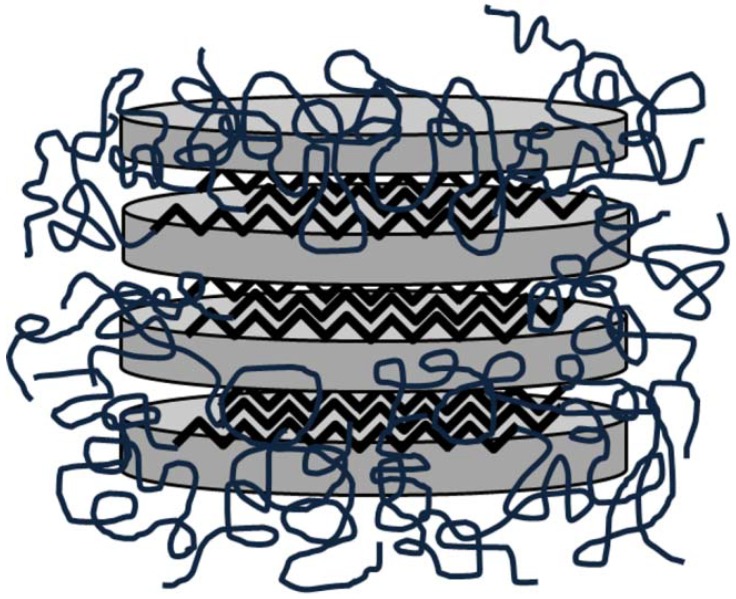
Schematic showing PI chains emanating from the Laponite^®^ platelets filled with PEO chains (“hairy” plates).

Gournis *et al.* investigated the morphology of two polymer-clay nanocomposites: Laponite^®^/PEO clay/homopolymer and Laponite^®^/PEO*-b-*polyisoprene (PEO-PI) clay block copolymer nanocomposites [164]. An adjustment of the layer spacing was observed for the Laponite^®^/PEO clay, a result of the elasticity provided by the intercalated polymer. In the case of Laponite^®^/PEO-PI clay, “hairy” plates were obtained, where the hydrophilic PEO was intercalated into the clay and the hydrophobic PI restrained the Laponite^®^ platelets from aggregation. A schematic of the “hairy” structure in the Laponite^®^/PEO-PI is presented in Figure 13. Michell *et al.* also investigated diblock copolymer-clay nanocomposites where the utilized copolymers were synthesized using PS, PI and dioctadecyl dimethyl ammonium-modified Laponite^®^ [165]. The visoeleastic data for the ordered and disordered states of the nanocomposites indicated minor changes compared to those of the unfilled polymer. This denoted that the addition of the silicates into the system had a negligible effect on the viscoelastic properties suggesting that a network structure was absent from these block copolymer-modified-Laponite^®^ nanocomposites. On the contrary, Agrawal *et al.* observed that the addition of Laponite^®^ platelets to an ABA triblock coplolymer, poly(lactide)-PEO-poly(lactide), had a significant impact on the rheological properties of the nanocomposites [166]. A particle-polymer network with enhanced elasticity was formed due to the PEO adsorption onto the silicate platelets.

Moreover, Liu *et al.* investigated the temperature-triggered gelation of Laponite^®^/poly(*N*-isopropyl acrylamide)/poly quaternarized *N,N-*dimethylaminoethyl methacrylate clay graft copolymer nanocomposites [167]. The addition of Laponite^®^ clay to the copolymer system had as a result: 1) a decrease of the gelation temperature of the nanocomposites; 2) a decrease of the critical concentration value of the copolymer needed for the gelation process. This observation was explained by the presence of interaction between Laponite^®^ and copolymer through electrostatic interactions and hydrogen bonding.

De Lisi *et al.* studied the thermodynamics of adsorption of triblock copolymers (PEO-PPO-PEO) at the interface of Laponite^®^ clay/solution when varying the hydrophilicity, the architecture and the molecular weight of the copolymer [168]. Calorimetric results and modeling kinetic data indicated that the architecture of the copolymers did not have a significant influence on the interactions between the Laponite^®^ platelets and the copolymers. However, keeping the ethylene oxide/propylene oxide ratio constant and varying the molecular weight of the copolymers, the authors observed that higher molecular weights resulted in an increase in the affinity for the Laponite^®^ clay. Moreover, the greater the hydrophilicity of the copolymer the bigger was the affinity for the Laponite^®^ surface. De Lisi *et al.* also investigated the effect of PEO-PPO-PEO triblock copolymers on aqueous Laponite^®^ clay dispersions by means of small-angle neutron scattering (SANS) [169]. SANS data indicated that the addition of PEO-PPO-PEO to the Laponite^®^ clay system retarded the gelation process in the time-window. Nelson *et al.* also performed SANS measurements on the adsorbed PEO-PPO-PEO triblock copolymers on Laponite^®^ clay [170]. The authors observed that the hydrophobic PPO segments were preferentially adsorbed at the surface of the Laponite^®^ platelets while the hydrophilic PEO were dangling in the solution. Later on, De Lisi *et al.* used the same triblock copolymer-Laponite^®^ systems for thermal and structural investigation [171]. The authors noticed that the enthalpy of melting of the nanocomposites was affected by the macromolecules radiating away from the Laponite^®^ clay while the macromolecules associated with the silicate did not have any contribution to the enthalpy of melting. The X-ray diffraction data of the nanocomposites indicated an exfoliated structure. Finally, De Lisi *et al.* also investigated the affinity toward an organic material, phenol, of the modified Laponite^®^ with PEO-PPO-PEO triblock copolymers [172]. The authors observed that the addition of copolymers to a phenol/Laponite^®^ solution led to the replacement of the organic material by the PEO-PPO-PEO. However, when the inversed copolymer (PPO-PEO-PPO) was used, the anchoring of phenol to the Laponite^®^ surface was enhanced.

### 4.6. Polyurethane/Montmorillonite Composites

Montmorillonite (MMT), a layered silicate clay, has been the focus of extended research for the preparation of polyurethane (PU)/clay nanocomposites. Due to its high aspect ratio and stiffness, MMT was found to enhance the thermal stability, morphology [7,173,174,175], mechanical and physical properties [7,176,177,178] of polymer/clay nanocomposites. Dan *et al.* used a melt processing technique as a way to improve the morphological and physical properties of PU/MMT clay nanocomposites [173]. The PUs were synthesized using 4,4`-diphenylmethane diisocyanate and 1,4-butanediol as hard segments and poly(tetramethylene oxide) glycol or poly(butylenes adipate) glycol as soft segments. The clay added to the polymer system was either pure MMT or modified MMT clay with a quaternary ammonium salt containing two –CH_2_CH_2_OH groups. The XRD and TEM images of the PU/clay nanocomposites indicated that the pure MMT had a very poor dispersion with thick agglomerated clay particles present in the nanocomposites while the modified MMT showed dispersion close to exfoliation. The behavior was caused by the formation of H-bonding between the carbonyl groups in PUs and the hydroxyl groups present in the modified MMT which resulted in a very good dispersion of the clay. A significant increase in the tensile properties of the nanocomposites was observed with the addition of both pure MMT and modified MMT to the system. However, the modified MMT increased the tensile modulus and stress to a greater extent than the pure MMT due to a better dispersion of the exfoliated silicate layers.

Furthermore, Dai *et al.* also synthesized PU/MMT nanocomposites via a solution-intercalation technique [7]. The sodium cations of the MMT clay were exchanged with organophilic alkyl-ammonium ions in order to improve the dispersity of the stiff nanolayers of the silicate clay into the polymer solution. Due to the presence of intercalated or exfoliated clay layers, the nanocomposites containing the modified MMT exhibited an increase in the tensile strength and elongation at break. A cationic-exchange reaction of the MMT was also performed by Lee *et al.* [174]. The XRD and TEM images of the PU/modified MMT nanocomposites indicated that the silicate platelets were exfoliated. Because of the high thermal insulation effect of the MMT clay, the PU/clay nanocomposites showed an increase in the thermal resistance. The tensile properties of the nanocomposites were also improved with the addition of the modified MMT. In addition, Cheng-Yang *et al.* synthesized PU/clay nanocomposites with very good mechanical and thermal properties by using a de-aggregated, solvent-swollen, organic modified MMT [177]. The modified MMT with 12-aminolauric acid was first de-aggregated into smaller size particles in DMF to prepare a stable suspension. Next, the suspension was mixed with the PU and highly exfoliated PU/clay nanocomposites were obtained, as indicated by TEM micrographs. The presence of the modified MMT into the polymer system decreased the mobility of the polymer chains which resulted in the retardation of the glass transition of the nanocomposites. The TGA measurements showed a great improvement of the thermal solubility of the nanocomposites with the addition of modified MMT. Moreover, an increase in the mechanical properties was observed. Tien *et al.* also used a swelling agent-modified MMT for the preparation of PU/clay nanocomposites [178]. The authors observed that with the increase of hydroxyl groups present in the swelling agent, the extent of intercalation of the modified MMT clay also increased. As a result, the tensile strength of the nanocomposites was dramatically improved. The behavior was attributed to the increase of the interfacial bonding between the PU molecules and the modified MMT containing the highest amount of hydroxyl groups.

### 4.7. Poly(ε-Caprolactone)/Montmorillonite Composites

Polymer clay nanocomposites with improved properties have been reported using poly(ε-caprolactone) (PCL) and MMT. Several methods have been employed in the past for the preparation of PCL/MMT nanocomposites, such as *in situ* [179,180] or bulk [181] polymerization, melt blending [182], and solution mixing [183]. Wu *et al.* used a solution mixing technique for the preparation of PCL/MMT nanocomposites [183]. A solution of MMT dispersed in deionized water and *N,N*-dimethylformamide was mixed with a PCL/*N,N*-dimethylformamide solution. The resulting polymer/clay nanocomposites showed an increase in the melting crystallization temperature, phenomenon explained by the presence of MMT which could suppress the mobility of the PCL segments. TGA measurements indicated that an increase in the MMT content led to enhanced thermal stability of the PCL/clay nanocomposites. Tarkin-Tas prepared PCL/clay nanocomposites making use of a different technique [179]. *In situ* ring opening polymerization (ROP) of ε-caprolactone was employed in the presence of an initiator, dibutyltin dimethoxide. The clay used in the process was MMT modified with 1-decyl-2-methyl-3-(11-hydroxyundecyl) imidazolium cation via an ion-exchange reaction. The hydroxyl groups on the long alkyl chain of the alkyl-imidazolium cation acted as initiators in the ROP of the ε-caprolactone and also as chain transfer agents. Variations in the molar ratio of monomers to hydroxyl groups raised the possibility of adjusting the molecular weights of the PCL to the desired value. XRD results indicated the formation of an intercalated morphology of the nanocomposites whereas the TEM images allowed the visualization of highly exfoliated clay platelets throughout the polymer matrix. The high mobility of the hydroxyl groups at the end of the long flexible alkyl chain was found responsible for inducing the exfoliation, due to a very good availability of the functional groups for the initiation of the ROP of ε-caprolactone. TGA measurements revealed that the clay did not have any effect on the degradation temperature of the nanocomposites. DSC data showed that the clay platelets could act as nucleating agents for the PCL crystallization. As a result, an increase in the mechanical properties of the PCL/clay nanocomposites was observed. Liao *et al.* also performed an *in situ* ROP for the preparation of nanocomposites [180]. However, instead of the conventional heating the authors used microwave irradiation which proved to have a greater monomer conversion and molecular weight and to be much faster. High degrees of exfoliated and/or intercalated structure were observed by TEM images. TGA measurements showed that the thermal stability of the nanocomposites was affected by both the clay and the molecular weights of the polymer.

Kiersnowski *et al.* explored a bulk polymerization technique for the preparation of PCL/clay nanocomposites [181]. Both pure MMT and MMT modified with hexadecyltrimethylammonium bromide (HTAB) or dodecylamine hydrochloride (DAC) were used in the process. The goal of the research was to understand how the presence of clay influences the intercalation of PCL. Formation of nanocomposites was observed for PCL/MMT and PCL/MMT-HTAB systems whereas microcomposites were obtained for PCL/MMT-DAC systems. Attractive interactions between polymer and neat silicate, as well as a relatively increased gallery height in MMT-HTAB, were found responsible for the formation of nanocomposites. In the case of PCL/MMT-DAC, DAC acts as a barrier for the incoming polymer to the silicate layer. Moreover, the gallery height of the MMT-DAC is too small and not enough volume is available to accommodate macromolecules. As a result, the growth of polymer chain stopped at early stages and microcomposites were formed.

At last, Yu *et al.* employed a melt blending technique to prepare hybrid poly(L-lactide)/PCL/organically modified MMT nanocomposites [182]. The authors observed that the addition of up to 5 wt% of modified MMT to polymers led to the formation of more exfoliated individual particles, improving in this way the thermal stability and crystalline abilities of poly(L-lactide)/PCL/modified MMT nanocomposites. The tensile strength, modulus and elongation at break were also enhanced with loading up to 3% of modified MMT, whereas higher contents of clay induced an increased brittleness in the material.

### 4.8. Polylactic Acid and/or Poly(Lactic-co-Glycolic Acid)/Montmorillonite Composites

Preparation of polymer clay nanocomposites using polylactide has also been previously reported [184,185,186,187,188,189,190]. Polylactide is a biodegradable polymer with excellent processability and physical properties. Despite some useful characteristics, the heat resistance, melt-viscosity and mechanical properties of polylactide are not satisfactory. To improve these properties, MMT clay and/or organically modified MMT were used among other fillers for the preparation of nanocomposites. Lee *et al.* combined an electrospinning process with a salt leaching/gas foaming method as a way to prepare modified MMT reinforced polylactide nanocomposite scaffolds with potential applications in the facile transport of metabolic nutrients, cell implantation and blood vessel invasion [187]. Dimethyl dehydrogenated tallow ammonium was employed as a cation for the modification of the MMT clay. The electrospun fibers resulted from the electrospinning process provided an interconnected nano-sized porous network. However, the porosity of the scaffolds was difficult to control especially when larger-sized pores were required. To obtain micro-sized pores, a salt leaching/gas foaming method was employed. NH_4_HCO_3_/NaCl salt particles scattered in the interlaminar layers of electrospun fibers sheets were mechanically kneaded and molded by compression. Next, the samples were immersed in hot water to generate gaseous ammonia and CO_2_ within the solidifying polymer matrixes. The resulting scaffolds presented dual porous structure with nano-sized and micro-sized pores as indicated by SEM micrographs. The incorporation of the modified MMT clay to the polylactide fibers increased the mechanical and biodegradable characteristics of the scaffolds.

Moreover, Urbanczyk *et al.* used *in situ* ROP in supercritical CO_2_ to synthesize polylactide/clay nanocomposites [189]. MMT modified with dimethyl (dehydrogenated tallowalkyl) ammonium cation (MMT1) or with bis (2-(hydroxyethyl) methyl) (tallowalkyl) ammonium cation (MMT2) were used to enhance the properties of the polylactide. The authors observed that the nature of the clay had an important role on the morphology of the nanocomposites synthesized by ROP of polylactide. The XRD and TEM images revealed that nanocomposites prepared with MMT1 had intercalated morphologies whereas those containing MMT2 revealed exfoliated morphologies. The hydroxyl groups present in the MMT2 clay could act as initiators and gave rise to polylactide chains grafted onto the clay surfaces resulting in an efficient delamination of the clay stacks. The delaminated polylactide/clay nanocomposites indicated a great improvement in both stiffness and toughness when compared with the unfilled polylactide matrix. Jiang *et al.* also obtained nanocomposites with improved properties when employing a melt extrusion technique to prepare polylactide/organically modified MMT nanocomposites [186]. The polylactide/MMT nanocomposites containing low concentration of MMT showed higher tensile strength and strain-at-break when compared with pure polylactide. This behavior was attributed to the presence of high degrees of chain orientation in the nanocomposites due to the shear amplification effect caused by the MMT platelets. Additionally, Gu *et al.* studied the linear and nonlinear shear rheological behaviors of polylactide/organophilic-MMT nanocomposites [185]. A master batch method was adopted for the preparation of the nanocomposites and dioctadecyl dimethyl ammonium bromide was employed for the modification of MMT clay. The authors observed that the linear viscoelastic properties of the melted nanocomposites displayed a solid-like rheological response (a result of strong friction between clay platelets) with increasing the clay content in the polylactide matrix. An increase of storage moduli, loss moduli and dynamic viscosities of the nanocomposites was also detected with the increase of the MMT content. The nonlinear viscoelastic results of the nanocomposites indicated that at low shear rates, the steady shear viscosities were much higher than the one of the pure polylactide and that the viscosity increased dramatically with the MMT content, whereas at higher shear rates the steady shear viscosities were lower than that of polylactide, as a result of the formation of shear-induced alignment of the dispersed MMT platelets.

Drug-loaded polymer/MMT nanoparticles have also been reported in the past [184,188,190]. For example, Dong *et al.* and Sun *et al.* prepared poly (D,L-lactide-co-glycolide)/MMT (PLGA/MMT) nanoparticles for oral delivery of paclitaxel, an anticancer drug [188,190]. An emulsion/solvent evaporation method was employed for the preparation of paclitaxel-loaded PLGA/MMT nanoparticles. The authors observed that the presence of MMT clay into the system could significantly increase the interactions between nanoparticles and cells due to the formation of London-van-der Vaals forces and H-bonds [190]. The presence of the strong interactions was essential to enhance the absorption of the drug-loaded nanoparticles. In vitro drug release studies of the loaded nanoparticles revealed an initial burst in the first day followed by a release of paclitaxel at a slow constant rate. It was observed that for the nanoparticles containing MMT, the initial burst was slightly reduced and the speed of drug release was increased when compared to the MMT free nanoparticles. Feng *et al.* also synthesized nanoparticles for oral delivery of anticancer drugs with docetaxel as a model drug [184]. Docetaxel-loaded polylactide-vitamin E d-α-tocopheryl polyethylene glycol 1000 succinate (TPGS) /MMT nanoparticles were prepared by a modified solvent extraction/evaporation method where TPGS was used due to its high emulsification effect as well as the high drug encapsulation efficiency.

### 4.9. Poly(Ethylene Oxide)/Montmorillonite Composites

Nanocomposites of poly(ethylene oxide) with montmorillonite have been extensively studied in the past decade [5,191,192,193,194]. It has been recently shown that the ionic strength in PEO-MMT nanocomposite gels, dictated by addition of various Li^+^ and Na^+^ salts, affects the extent of internal orientation of such systems when subjected to elongational flow [194]. For example, Li based salts triggered the formation of stronger polymer clay networks, owing to the ability of Li^+^ ions to screen water/PEO hydrogen bonding to a greater extent than Na^+^ ions. The disruption of hydrogen bonding promoted the coordination of PEO oxygens to the metals from the surface of the MMT platelets. Additionally, it was found that for salts of the same metal (e.g. Li^+^), the bulkiness of the counter ion could also affect the extent of orientation in such processes. For example gels containing the bulky SO_4_^-^ counterion could orient easier than gels containing the relatively small Cl^-^ anion [194].

**Figure 14:**
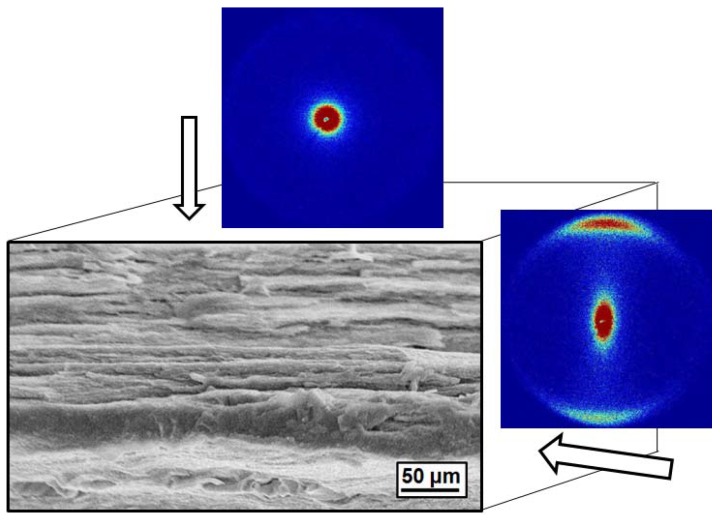
Schematic of a PEO-MMT film, containing Li_2_SO_4_, that was prepared through a layer-by-layer approach. The exposed freeze-fractured edge was analyzed via SEM and a micrograph is displayed. Additionally 2D SAXS patterns are presented for the cases when the X-ray beam is projected on the edge and on the top surface of the film.

Furthermore, in films prepared from such PEO-MMT gels it was observed that the nanoscopic MMT platelets oriented with their surface parallel to the plane of the film [193]. The strength of the network in PEO-MMT gels appeared to have a significant impact on the morphology of films prepared via a layer-by-layer approach, where a lower gel viscosity resulted in finer polymer-clay layers in the films. Additionally, it was observed that the strength of the network in the nanocomposite gels containing various salts was inversely proportional to the fraction of crystalline polymer, and ultimately to the storage modulus, in the resultant nanocomposite films [193]. Figure 14 presents a SEM micrograph obtained from a PEO-MMT film containing Li_2_SO_4_. Two 2D SAXS patterns are additionally presented for the cases when the X-ray beam was projected perpendicular to the edge and to the top surface of the film. Highly oriented layers resulted from the layer-by-layer preparation method can be observed in the SEM micrograph. In addition, the anisotropic SAXS pattern observed from the edge of the film coupled with the isotropic pattern seen when the X-ray beam is projected from the top clearly indicate that the MMT platelets are oriented with the surface parallel to the plane of the film.

### 4.10. Polypropylene/Montmorillonite Composites

Polypropylene (PP) is one of the most widely used polyolefin polymers because of its low density, low cost and excellent chemical resistance. When organically modified MMT is dispersed in the polymer matrix an enhancement in mechanical properties, heat distortion temperature and a decrease in flammability of the corresponding nanocomposites is typically observed. Sun *et al.* synthesized such nanocomposites through a melt-mixing method by using MMT modified with tetraethylene pentamine and vinyl triethoxy silane as a coupling agent [195]. The modification of the hydrophilic MMT to a hydrophobic MMT was necessary to increase the compatibility of the clay with the PP while the coupling agent was used to enhance the interlayer spacing of the MMT. The authors observed an improvement in the mechanical and thermal properties of the nanocomposites, as a result of a good dispersion of organomodified MMT in the polymer matrix, as indicated by TEM micrographs and XRD patterns.

Rohlmann *et al.* investigated the linear viscoelasticity behavior of PP/organophilic MMT nanocomposites prepared via melt-mixing [196]. The MMT was modified with dimethyldialkyl ammonium halide to ensure a better compatibility with the polymer which facilitated the exfoliation and dispersion of the clay platelets in the polymer. However, a complete exfoliation was not achieved for the PP/ organophilic MMT systems. The addition of PP grafted with maleic anhydride (a coupling agent) resulted in a complete exfoliation of the clay platelets. Dynamic strain sweep measurements of the nanocomposites indicated a linear viscoelastic behavior that was found to depend on the concentration of the clay. The maximum strain of linear viscoelastic behavior is reduced with the increase in the amount of the exfoliated platelets suggesting that the linear viscoelastic region was sensitive to the composite structure. Moreover, the annealed nanocomposites indicated a solid-like rheological behavior, a result of percolated network superstructure formed due to physical interaction between the MMT platelets.

**Figure 15:**
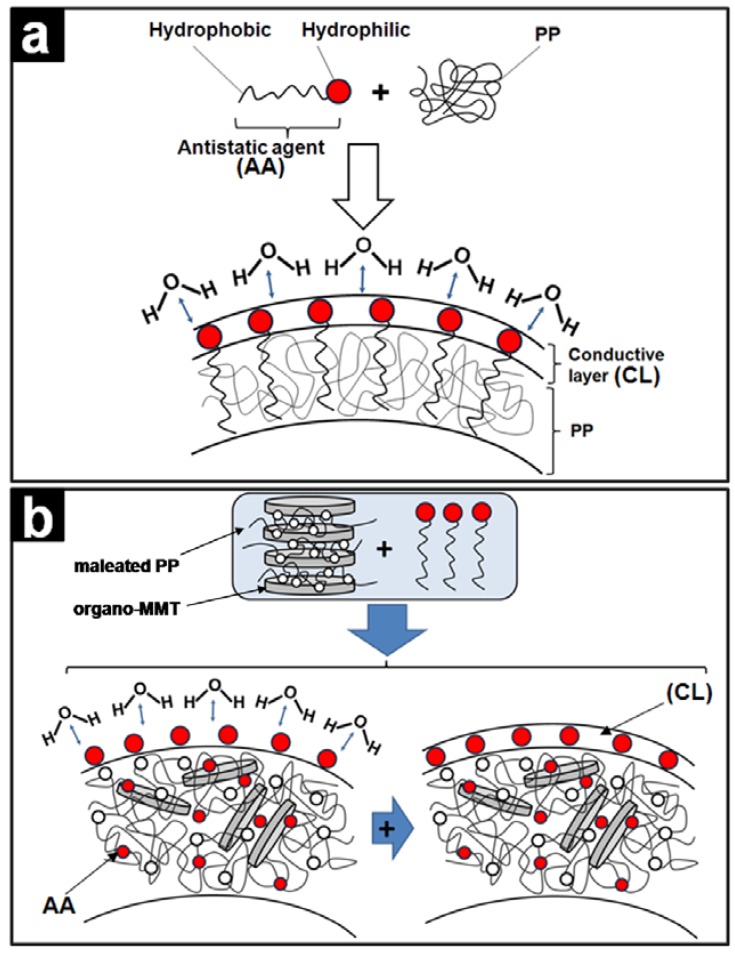
Schematic depicting possible interaction mechanisms between either PP and AA (a), or between PP, AA and organo-MMT (b).

Unlike Rohlmann *et al.* [196], Qin *et al.* observed a nearly exfoliated dispersion of clay platelets in the polymer matrix for the nanocomposites containing PP/ maleated PP/ modified MMT with diocta-decyldimethyl ammonium chloride [197]. The photo-oxidative behavior of these nanocomposites was investigated and it was detected that an exposure to UV radiation resulted in a faster photo-oxidative degradation than that of pure PP. Photoresponsive groups introduced in the system with the addition of modified MMT and maleated PP were responsible for the increase of photo-oxidative degradation of the PP matrix. Nowacki *et al.* prepared a similar system of isotactic PP/ maleated PP/ organically modified MMT, with the difference that the modification of MMT was performed with octadecyl amine [198]. The authors investigated the nucleation of spherulites in the nanocomposites under shear. It was observed that shear-induced crystallization of the films obtained by compression molding augmented the nucleation density when compared with samples crystallized in static conditions. A decrease in the spherulite sizes was also noted. A possible explanation for the behavior could be the effect of increased local stress and orientation of polymer chains under shearing. Higher shear rates also resulted in an increase of the nucleation intensity.

Finally, Chow *et al.* incorporated in the PP/ maleated PP/ organo-MMT nanocomposites an antistatic agent (AA) based on polyamide/polyether block amide [199]. The authors observed that the presence of the AA had minor effects on the tensile strength, tensile modulus, elongation at break and impact strength of the nanocomposites. However, a better wettability and interfacial interaction between organo-MMT and PP matrix was achieved with the addition of the AA. The surface resistivity was also investigated and it was observed that at lower concentration of AA (3–6 wt%) the nanocomposites exhibited an insulator behavior while at higher concentration of AA (9 wt%) an antistatic behavior was achieved. A possible mechanism for the interaction and intercalation of PP/ maleated PP/ organo-MMT nanocomposites is depicted in Figure 15.

## 5. Conclusions

Metal-polymer nanocomposite materials are becoming more popular for applications requiring low cost, high metal surface areas. Catalytic systems seem to be the most prevalent application for a wide range of metals used in polymer nanocomposites, particularly for metals like Pt, Ni, Co, and Au, with known catalytic activities. Electrically active devices obtained by embedding metal nanoparticles in conductive polymer matrices are also a popular set of applications for these types of materials. Worthy to be mentioned are optically active devices, along with a wide variety of Fe, Co and Ni based magnetically active products. From a muscle like structure to bioactive nanocomposites, these materials deliver enhanced properties over the original components, allowing for a great expansion their usage envelope.

From a synthetic perspective, today’s researchers have developed highly specific pathways for obtaining very well tailored properties. In addition, very cost effective and generally applicable synthesis pathways have been designed. Such methods are well suited for a large variety of metals and deliver nanoscale metal-polymer composite materials with significantly improved properties over the starting materials. Some of these methods are based on unique chemical bonds and/or treatments that help compatibilize metal surfaces with the organic matrixes; others simply utilize steric effects like mechanical wrapping of the polymers around metal nanospheres to obtain a metal-polymer nanocomposite product, while other yet use compounding methods to mechanically insert metal particles in polymer substrates. There seems to be a trend of preparing these nanocomposites “tailor-made” for a specific application, rather than customizing a universal product. Even though the great majority of the research described here presents a synthetic method of some sort, there are some papers published on modifications on premade metal-polymer nanocomposite. Overall, metal-embedded polymer nanocomposites bring a whole new level of possibilities to the world of engineered materials, enhancing already existing properties within the starting materials, as well as creating a new set of properties found only in the composite structures.

Furthermore, following the recent developments in the fields of nanotechnology, CNT and clay reinforced polymer composites received tremendous attention in the past decades, and currently occupy elite places among the most studied polymer composites. Some of the most frequently utilized techniques to prepare polymer/CNT and/or polymer/clay nanocomposites include melt mixing, solution casting, electrospinning and solid-state shear pulverization. Literature reports indicate that similar to the solution casting method, electrospinning and solid-state shear pulverization are techniques completely benign to the various polymer matrixes. On the other hand, prolonged mixing times, which are often needed to achieve good filler dispersion when the melt compounding technique is used, regularly lead to extensive polymer degradation. Regardless of the technique utilized, some reports suggest that a good dispersion of CNTs and clays in polymer matrixes and formation of percolated networks sometimes may not be synonymous, especially when the dispersed fillers are aligned predominantly in one direction. When not functionalized, often times clays and CNTs can only be dispersed in polymer matrixes as microscale aggregates because of their poor affinity for the polymers and increased tendency for agglomeration. The interfacial adhesion between clays and/or CNTs and the polymer matrixes is often times improved by functionalizing the filler, fact that allows for a homogeneous nanoscale-dispersion of the reinforcing agent within the matrixes. Other times various surfactants (such as anionic surfactants) may be employed to achieve an extensive dispersion of the fillers. Some of the chemicals (covered in this review) utilized to functionalize CNTs include maleic anhydride, octadecylamine, polyethylene glycol, poly(hexamethylene adipamide), poly(bisphenol-A-co-epichlorohydrin), and various acids. On the other hand, some of the chemicals utilized here to functionalize clays include γ-methacryloxypropyltrimethoxysilane, cetyltrimethyl-ammonium bromide, PEO-PPO-PEO copolymers, 12-aminolauric acid, poly(ε-caprolactone), dimethyl (dehydrogenated tallowalkyl) ammonium and/or bis (2-(hydroxyethyl) methyl) (tallowalkyl) ammonium cations, etc. When properly dispersed within the matrixes, CNTs and clays have the ability to improve the properties of the resultant materials several orders of magnitude relative to the unfilled polymers. The enhanced properties may include tensile behavior, strength, toughness, stiffness, electrical and thermal conductivity and crystallization kinetics. An example of the latter case is that ordered nanotubes can act as nucleating templates for the growth of oriented crystalline superstructures. The presence of CNTs often leads to an increase in the melting/crystallization temperature of the polymer matrix, as it is the case for isotactic polypropylene. However, for some other polymers, such as poly(ethylene oxide), the presence of nanotubes leads to a decrease of the melting/crystallization temperature. In addition, some studies suggest that the presence of CNTs do not strongly impede the chain motion of polymer matrix.
