# Erratum: Stefanescu, E.A.; Daranga, C.; Stefanescu, C. Insight into the Broad Field of Polymer Nanocomposites: From Carbon Nanotubes to Clay Nanoplatelets, via Metal Nanoparticles. *Materials* 2009, *2*, 2095–2153

**DOI:** 10.3390/ma9060459

**Published:** 2016-06-13

**Authors:** Materials Editorial Office

**Affiliations:** Klybeckstrasse 64, CH-4057 Basel, Switzerland

It has been brought to our attention that Laponite^®^ is a trademark of BYK Additives, however the trademark symbol is missing in [1]. We apologize for an inconvenience caused by this omission. The symbol will be added to the online version of the article.
